# Cassava yield traits predicted by genomic selection methods

**DOI:** 10.1371/journal.pone.0224920

**Published:** 2019-11-14

**Authors:** Luciano Rogério Braatz de Andrade, Massaine Bandeira e Sousa, Eder Jorge Oliveira, Marcos Deon Vilela de Resende, Camila Ferreira Azevedo

**Affiliations:** 1 Department of Plant Science, Universidade Federal de Viçosa, Viçosa, Minas Gerais, Brazil; 2 Center of Agrarian, Environmental and Biological Sciences, Universidade Federal do Recôncavo da Bahia, Cruz das Almas, Bahia, Brazil; 3 Embrapa Mandioca e Fruticultura, Cruz das Almas, Bahia, Brazil; 4 Department of Forestry Engineering, Universidade Federal de Viçosa, Viçosa, Minas Gerais, Brazil; 5 Embrapa Florestas, Colombo, Paraná, Brazil; 6 Department of Statistics, Universidade Federal de Viçosa, Viçosa, Minas Gerais, Brazil; Shanghai Institutes for Biological Sciences, CHINA

## Abstract

Genomic selection (GS) has been used to optimize genetic gains when phenotypic selection is considered costly and difficult to measure. The objective of this work was to evaluate the efficiency and consistency of GS prediction for cassava yield traits (*Manihot esculenta* Crantz) using different methods, taking into account the effect of population structure. BLUPs and deregressed BLUPs were obtained for 888 cassava accessions and evaluated for fresh root yield, dry root yield and dry matter content in roots in 21 trials conducted from 2011 to 2016. The deregressed BLUPs obtained for the accessions from a 48K single nucleotide polymorphism dataset were used for genomic predictions based on the BayesB, BLASSO, RR-BLUP, G-BLUP and RKHS methods. The accessions’ BLUPs were used in the validation step using four cross-validation strategies, taking into account population structure and different GS methods. Similar estimates of predictive ability and bias were identified for the different genomic selection methods in the first cross-validation strategy. Lower predictive ability was observed for fresh root yield (0.4569 –RR-BLUP to 0.4756—RKHS) and dry root yield (0.4689 –G-BLUP to 0.4818—RKHS) in comparison with dry matter content (0.5655 –BLASSO to 0.5670 –RKHS). However, the RKHS method exhibited higher efficiency and consistency in most of the validation scenarios in terms of prediction ability for fresh root yield and dry root yield. The correlations of the genomic estimated breeding values between the genomic selection methods were quite high (0.99–1.00), resulting in high coincidence of clone selection regardless of the genomic selection method. The deviance analyses within and between the validation clusters formed by the discriminant analysis of principal components were significant for all traits. Therefore, this study indicated that i) the prediction of dry matter content was more accurate compared to that of yield traits, possibly as a result of the smaller influence of non-additive genetic effects; ii) the RKHS method resulted in high and stable prediction ability in most of the validation scenarios; and iii) some kinship between the validation and training populations is desirable in order for genomic selection to succeed due to the significant effect of population structure on genomic selection predictions.

## Introduction

Cassava (*Manihot esculenta* Crantz) is of great worldwide importance as a food security crop. In Brazil, it is cultivated all over the country in adverse and quite contrasting conditions with respect to water and nutrient availability, and in many cases where of commodities crops is not recommended [1]. Despite the economic and social importance of cassava, the use of quantitative genetics and plant breeding knowledge to develop this crop was initiated only a few decades ago. Thus, great progress is still needed in germplasm development for cassava compared to other globally important crops such as corn, rice, soybean and potato.

In general, breeding programs use quantitative and population genetics knowledge to generate and select new genotypes with higher yields than the commercial varieties available for cultivation [2]. In cassava, breeders use vegetative propagation to their advantage, promoting hybridizations between contrasting parents and aiming to select superior F_1_ individuals [3–5]. Once a superior genotype is identified, it can be clonally propagated and evaluated in field conditions, i.e., clonal evaluation trials. However, the root system from plants derived from sexual seeds often undergo changes of shape and size through seedlings to clonal propagation, and the clone selection in early stages of cassava breeding programs, such as the seedling stage can be inefficient due to no linear relationship for fresh root yield between seedlings and advanced stages of clonal propagation [6]. This limitation affects the selection gain per unit of time, since the identification of new varieties is also linked to their propagation rate over the evaluation years. This affects the efficiency of the cassava breeding program, especially due to the long time needed to develop new varieties and the high costs of phenotyping across various locations and years of cultivation.

The ability to select individuals in early stages, thereby maximizing genetic gains and developing new varieties more quickly, is a key goal for several breeding programs. There are currently great expectations for the development of genomic selection applications to make this possible [7–8], since genomic selection allows the selection and early recombination of promising genotypes and families without phenotypic evaluation. This approach is even more valuable when phenotypic selection is expensive and/or inefficient [9], as has been observed in the seedling phase in cassava, in which heritability for important agronomic traits is low.

According to Resende et al. [10], genomic selection efficiency can be estimated by the selection accuracy, which depends on the trait heritability, number of loci and distribution of their effects, training population size, effective population size and the number and distribution of markers in the genome of the species. However, only the last three factors can be controlled, aiming for greater efficiency and accuracy regardless of the genomic selection method used. According to Isidro et al. [11], population structure is a consequence of having different population genetic histories composing a larger population; these distinct subpopulations could have differences in allele frequencies for many polymorphisms throughout the genome, as fixed alleles resulting from different selection pressures or directions. The population structure can mimic association signal, increasing the number of false positives or to missed real effects in association studies [12]. Oliveira et al. [3] demonstrated the importance of designing the training sets for genomic selection in cassava in order to achieve a highly efficient selection model, since, in general, populations with complex population structures tend to have lower accuracy [13].

Genomic selection models make it possible to obtain the genomic estimated breeding values (GEBVs), which are the genetic values predicted by genomic selection model for each individual. The prediction of GEBVs can be performed using several methodologies, including RR-BLUP, G-BLUP, BayesA, BayesB, BayesCπ, BayesDπ, Lasso, BLASSO, IBLASSO, and RKHS [1, 14–18]. However, the proportion of the genetic variance explained by markers differs according to the prediction method used, since each method has different assumptions with respect to the markers’ distribution effects, the selection of covariates and/or the genetic variances and covariances matrix. Different combinations of these assumptions modify the genetic variation explained by markers, which reflects directly on the accuracy. Different accuracies between the genomic selection methods can occur in real data due to trait inheritance, which can alter the genetic variation due to the number of genes and the epistatic and non-epistatic allelic relationships [19].

In general, there is great expectation for the adoption of genomic selection in both the public and private sectors world-wide [20], given the possibility of increasing the efficiency and productivity of plant breeding [20–25]. As reported by Oliveira et al. [3], reduction of the breeding cycle allows higher selection gains per unit of time, even with low to medium accuracy.

By shortening the selective cycle by one-year, genomic selection was more efficient than phenotypic selection by 4.6% for fresh root yield, and these gains may be even higher (73%) if the cassava selection cycle can be reduced from 4–5 years to 2 years [3]. According to Heffner et al. [8], reducing the breeding cycle allows breeders to concentrate resources in promising individuals in new breeding cycles or to advance in the selection trials of new varieties. However, according to these authors, low accuracy and high initial cost, especially in the implementation phase of this method, may reduce interest in genomic selection.

Recently, there have been several efforts to implement genomic selection in cassava breeding programs. The results have shown potential for increased genetic gains for several traits, especially resistance to cassava mosaic disease (CMD), dry matter content and fresh root yield [4–5, 26–27]. For CMD resistance, adopting genomic selection through two recurrent selection cycles in two years increased the allelic frequency of resistance from 44 to 66% [27]. Commonly, the development of a new cassava cultivar takes eight to ten years [1–4]. Therefore, there is a great expectation in the use of genomic selection in this species to speed up the development of new varieties.

This work evaluated the efficiency of different genomic selection methods for clone selection for yield traits, such as fresh root yield, dry root yield and dry matter content. These methods were tested in different scenarios of population structure with the aiming to estimate and infer the effect of population structure on the efficiency and consistency of genomic selection methods. The results for early selection using genomic selection are discussed, as well as the implications of population structure on genomic prediction.

## Materials and methods

### Training population

The training population consisted of 888 cassava accessions obtained from the Cassava Germplasm Bank of Embrapa Cassava and Fruits (Cruz das Almas, Bahia, Brazil). They included 835 landraces and 53 improved varieties of which, 190 were characterized as sweet cassava, 125 as intermediary cyanide content, 557 as bitter cassava and 16 accessions were not yet classified for this trait. All 26 Brazilian states were represented by at least one genotype. The genotypes were evaluated in Cruz das Almas and Laje, both in the State of Bahia, Brazil, totaling 21 trials distributed in six years from 2011 to 2016.

### Phenotypic data

The 21 trials included two randomized complete block design (RCBD) trials and 19 augmented block design (ABD) trials with 16 plants per plot. Three replicates were used in the CRBD, while in the ABD, 10 to 22 replicates of the common checks were used, with equal distribution of accession numbers per block. Improved clones (9602–02, 9607–07, 9824–09, 9655–02) and cultivated varieties (BRS Dourada, BRS Gema de Ovo and BRS Novo Horizonte) were used as controls in different field trials.

In most experiments, 15–20 cm stem cuttings were planted in double lines during the region’s rainy season (May to July). The spacings between rows and plants were 0.9 and 0.8 m, respectively. All recommended cassava cultural practices were adopted. The plants were harvested between 11 and 12 months after planting. The traits measured to estimate the genomic selection efficiency were fresh root yield (t.ha^-1^), dry matter content in roots (%) according to Kawano et al. [28], and dry root yield (t.ha^-1^), estimated by the product of dry matter content in roots [28] and fresh root yield. Dry matter content was estimated by root specific gravity, determined from the weights in air and in water [28]. Due to genotype and repetition imbalance between trials, the BLUP and deregressed BLUP were obtained for each genotype and the phenotypic heritability for each trait. The BLUPs were obtained by the following mixed linear model: *y*_*ijl*_ = *μ* + *c*_*i*_ + *β*_*j*_ + *r*_*l*(*j*)_ + *ε*_*ijl*_, where *y*_*ijl*_ is the vector of phenotypic observations; *c*_*i*_ is the clone random effect with $c_{i}\sim N(0,\sigma_{i}^{2})$; *β*_*j*_ is the combination of location and year, assumed as fixed effect; *r*_*j(l)*_ is the replication nested within location and year, assumed as random effect with $r_{j(l)}\sim N(0,\sigma_{r}^{2})$; and *ε*_*ijl*_ is the residual with $\varepsilon_{ijl}\sim N(0,\sigma_{e}^{2})$. The deregressed BLUPs were estimated as follows: $deregressed\;BLUP=\frac{BLUP}{1-\frac{PEV}{\sigma_{i}^{2}}}$ [29], where PEV is the prediction error variance of each clone and $\sigma_{i}^{2}$ is the clonal variance component. The package *lme4* [30] of R 3.3.3 software [31] was used to obtain the BLUPs and deregressed BLUPs for each clone.

### Genotyping and SNPs quality control

The DNA was extracted following the cetyltrimethylammonium bromide (CTAB) protocol of Doyle and Doyle [32]. To evaluate the DNA’s integrity and standardize its concentration, 1.0% (w/v) agarose gels were stained with ethidium bromide (1.0 mg L^-1^) and visually compared with a series of phage Lambda DNA (Invitrogen) concentrations. The DNA samples were sent to the Genomic Diversity Facility at Cornell University (http://www.biotech.cornell.edu/brc/genomic-diversity-facility) for Genotyping by Sequencing (GBS) [33]. Individual DNA samples were digested with *Ape*KI restriction enzyme, barcode ligated and multiplexed into 95-plex libraries [33]. Library sequencing was performed on an Illumina HiSeq2500 platform. Subsequent sequence reads were demultiplexed and aligned to the cassava reference genome v.6 [34] using BWA [35]. SNP calling was performed using the TASSEL GBS pipeline [36]. A total of 72,023 SNPs distributed across all 18 cassava chromosomes was obtained. The genotypic data were selected considering a minimum call rate of 90% and the missing markers were then imputed using Beagle 4.1 software [37]. Finally, the SNPs with a minor allele frequency of at least 5% were selected. After the marker quality control, 48,655 SNPs were selected for the prediction. The GBS data are available through the Cassavabase (https://www.cassavabase.org/). The multilocus heterozygosity of individual and the observed heterozygosity (Ho) for SNP loci are presented in a supplementary material (S1 and S2 Tables). The pattern of LD for each chromosome was analyzed and all pairwise LD combinations (r^2^) were estimated (S1 Fig).

### Discriminant Analysis of Principal Components (DAPC)

The Discriminant Analysis of Principal Components clustering method [38] with an island migration model was used to infer meaningful clusters in the cassava germplasm, which were then used to evaluate the effect of population structure on the efficiency and consistency of genomic selection methods. The population structure of the 888 accessions was estimated via discriminant analysis of principal components using the coefficients of relationship due to identity by state [39]. After defining the number of groups, the main component analysis axes that explained more than 85% of the total variance of the data were maintained in the analysis (S2 Fig). Only the first four discriminant functions were used by the migration model to estimate the population structure, since they account for more than 94% of all variation between the accessions (S3 Fig). The population structure chosen was composed of five clusters with high accession discrimination capacity between clusters. This analysis was carried out using the *adegenet* package [40] from R 3.3.3 software [31].

### Genomic selection methods

The genomic selection methods were chosen based on different statistical assumptions as well as demonstrated success in accurately predicting GEBV in different crops. The additive models tested were RR-BLUP, G-BLUP, RKHS, BayesB, and BLASSO. BayesB, BLASSO and RR-BLUP shared the following model: *y*_*d*_ = *Jμ* + *Zβ* + *ε*, where *y*_*d*_ is the deregressed BLUPs vector; *μ* is the general mean; *β* is the allelic substitution effect vector; *ε* is the residual effect vector; and *J* and *Z* are the incidence matrices for *μ* and *β*, respectively. G-BLUP and RKHS followed the corresponding model: *y*_*d*_ = *Jμ* + *Xβ* + *ε*, where *y*_*d*_ is the deregressed BLUPs vector; *μ* is the general mean; *g* is the genetic individual effect vector; *ε* is the residual effect vector; and *J* and *X* are the incidence matrices for *μ* and *g*, respectively. These models differ in the distribution of marker effects and covariate selection (markers). RR-BLUP, G-BLUP and RKHS assume a normal distribution for the marker effects. RR-BLUP uses the *Z* marker matrix in the indirect prediction of GEBVs [7], while G-BLUP accounts for additive effects via the genomic relationship matrix (*G*), and RKHS accounts for both additive and non-additive effects using the Gaussian kernel matrix (*K*). These matrixes were estimated by the equations $G=\frac{ZZ\prime }{2\sum p_{i}(1-p_{i})}$ and $K=exp(\frac{-hD}{median(D)})$, where *Z* is the codified markers matrix (-1, 0, and 1), *p* is the major allele frequency of marker *i*, *h* is the reduction coefficient to *K* values, *h* is equal to 1, and *D* is the Euclidean distance of codified markers matrix [41,42]. The distribution of marker effects for the Bayesian methods is t-student and exponential for the BayesB [7,16] and BLASSO [17] models, respectively. Moreover, the BayesB method has covariate selection due to the estimated parameter *π*, which is the prior probability that marker *i* has a nonnull effect on the trait. The RR-BLUP method was carried out using the *rrBLUP* package [23], while G-BLUP and RKHS were carried out with the *sommer* package [21], and the BayesB and BLASSO methods with the *BGLR* package [24]. All of these packages are available in R 3.3.3 software [31]. For BayesB and BLASSO convergence, 10,000 Markov Chain Monte Carlo (MCMC) iterations were used, with a burn-in of the first 2,000 MCMC iterations and a sampling interval (thinning) of 10. All of the MCMC residual variances were obtained to evaluate the convergence diagnosis of the BayesB and BLASSO methods.

### Cross validation strategies

**Fig 1:**
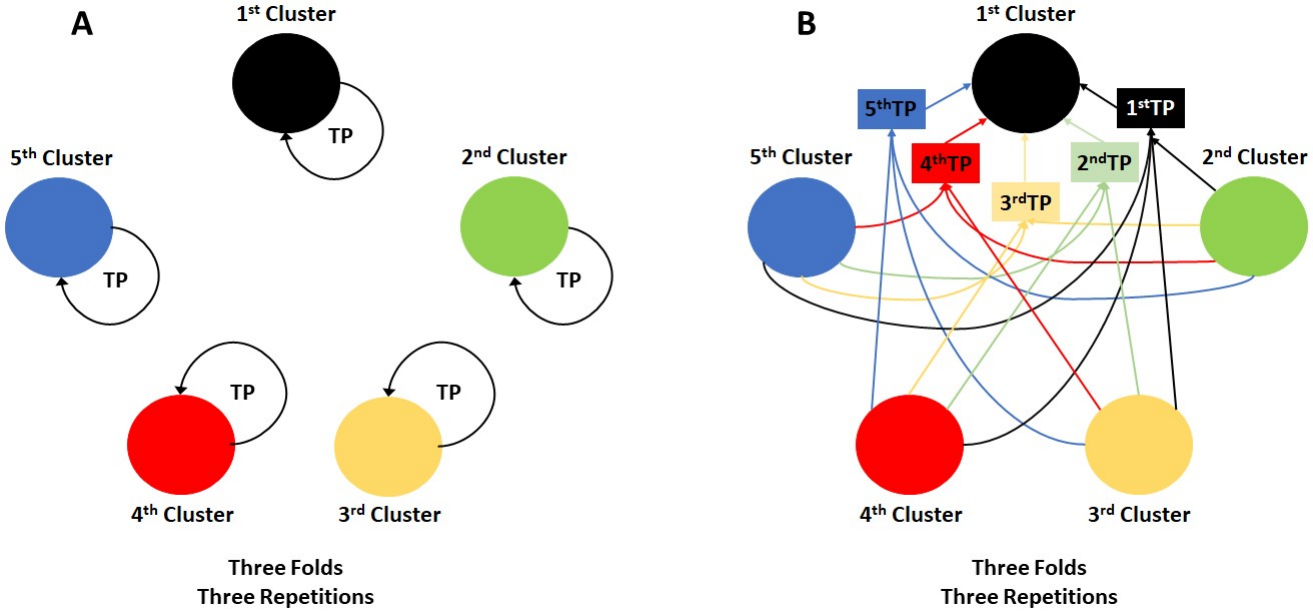
Cross validations performed to estimate the effect of population structure in genomic selection. **A**. 2^nd^ cross validation strategy–Within-cluster cross validation formed by discriminant analysis of principal components (DAPC); **B**. Illustration of between-clusters cross validation for DAPC cluster 1: 3^rd^ cross-validation strategy–cross validation with training population composed of a third part of DAPC clusters 2–5 and validation in a third part of DAPC cluster 1 (**1**^**st**^**TP**); 4^th^ cross-validation strategy–cross validation with the training population composed by a third part of three of the DAPC clusters (**2**^**nd**^, **3**^**rd**^ **4**^**th**^, **and 5**^**th**^ **TPs**). Therefore, the first training population (**1**^**st**^ **TP**) is formed from the four remaining DAPC clusters (3^rd^ cross validation strategy), while the second, third, fourth and fifth training populations (**2**^**nd**^, **3**^**rd**^ **4**^**th**^ **and 5**^**th**^ **TPs**) were each created from only three of those four DAPC clusters (4^th^ cross validation strategy).

To estimate the efficiency and consistency of the genomic selection methods, different cross-validation strategies were performed. In the first strategy, cross-validation was performed using three replicates with five folds each, independently of the population structure. Three other cross-validation strategies were performed considering the population structure effect, both with three folds and three replicates. The second cross-validation strategy was conceived considering the validation and training populations composed by the same DAPC cluster (Fig 1A). The last two cross-validation strategies were performed to prevent the presence of clones from the same cluster in training and validation populations. So, the following cross-validation strategies were used: for the third cross-validation strategy, the training populations were composed of accessions from all clusters, except the cluster used for validation. For example, in the validation of the first cluster, the accessions belonging to the second, third, fourth and fifth clusters were used for training (first training population–TP–Fig 1B). Finally, for the fourth cross-validation strategy, the training populations were composed of accessions from only three clusters, excluding the cluster used for validation and one of the four remaining clusters. For example, in the validation of the first cluster, the accessions belonging to clusters 3, 4 and 5 were used for training in one scenario (second TP); and clusters 2, 4, and 5 (third TP); 2, 3, and 5 (fourth TP); and 2, 3, and 4 (fifth TP) in the other scenarios (Fig 1B).

All of the genomic prediction methods had the same scenarios of training population predicting a validation population. This was ensured by using the “set.seed()” function of R 3.3.3 software [31].

Cross-validation was used to estimate the following parameters: i) predictive ability $\left(r_{\hat{y}y}=COR({\hat{GEBV}}_{Val},y_{Val})\right)$, where ${\hat{GEBV}}_{Val}$ was the genomic estimated breeding values (GEBVs) of the validation population and *y*_*Val*_ was the BLUPs from the validation population; ii) bias $\left(b=COV({\hat{GEBV}}_{Train},y_{Train})/\sigma_{{\hat{GEBV}}_{Train}}\right)$, where ${\hat{GEBV}}_{Train}$ was the GEBVs of the training population, *y*_*Train*_ was the deregressed BLUPs from the training population, and $\sigma_{{\hat{GEBV}}_{Train}}$ was the GEBVs standard deviation of the training population; iii) prediction accuracy $\left(r_{\hat{y}g}=r_{\hat{y}y}/h_{phen}\right)$, where *h*_*phen*_ is the root square of phenotypic trait heritability; and iv) and genomic heritability $\left(h_{gen}^{2}=\sigma_{\hat{g}}^{2}/\left(\sigma_{\hat{g}}^{2}+\sigma_{e}^{2}\right)\right)$, where $\sigma_{\hat{g}}^{2}$ was the genomic variation and $\sigma_{e}^{2}$ was the residual variation.

### Convergence diagnostics

The Bayesian methods, BayesB and BLASSO, were evaluated via the Raftery and Lewis diagnosis [43] implemented in the *coda* package [44] from R 3.3.3 software [31]. This diagnostic evaluates the Bayesian analysis convergence using residual variances of the MCMC iterations to estimate the number of MCMC iterations required for each cross validation.

### Deviance analysis and mean test

The deviance analyses were performed to evaluate the effects of the genomic selection methods and population structure on genomic prediction. Genomic selection methods were assumed as fixed effects for predictive ability ($r_{\hat{y}y}$) and bias (*b*) estimates for dry matter content, fresh root yield and dry root yield. These analyses were performed using the *lme4* package [30] from R 3.3.3 software [31]. The following model was used to estimate the methods’ efficiency in the 1^st^ cross validation strategy: *y* = *s* + *m* + *e*, where *y* is the dependent variable, being either predictive ability or bias; *s* is the cross validation random effect; *m* is the genomic selection methods fixed effect; and *e* is the residual. To estimate the effect of population structure on genomic selection efficiency and consistency, the following model was used in the 2^nd^, 3^rd^, and 4^th^ cross validation strategies: *y* = *s* + *p* + *m* +*e*, where *y* is the dependent variable, being either $r_{\hat{y}y}$ or *b*; *s* is the cross validation random effect; *p* is the interaction random effect between the training and validation populations; *m* is the genomic selection methods fixed effect; and *e* is the residual. The means of the different genomic prediction methods were submitted to Tukey’s comparison test as implemented in the *emmeans* package [45] from R 3.3.3 software [31].

### Cohen’s Kappa coefficient between GEBVs from different genomic selection methods

The Cohen’s Kappa coefficient [46] was estimated from the coincidence selection of genomic selection methods, which was performed between the different prediction methods with a selection proportion (SP) ranging from 1 to 30%. The coincidence selection was performed using a binary code; the selected and unselected individuals received codes of 1 and 0, respectively. The Kappa coefficient and the coincidence selection were calculated using R 3.3.3 software [31].

## Results

### Estimates of genomic selection efficiency

The BayesB and BLASSO models were evaluated for convergence in all cross validations. Ten thousand MCMC iterations proved to be sufficient for both convergence methods in all cross validations, according to the Raftery and Lewis method [43]. All of the genomic prediction methods showed higher genomic heritability estimates (Table 1) than the phenotypic heritability for all traits (0.337, 0357, and 0.545, for fresh root yield, dry root yield and dry matter content, respectively). The BayesB, BLASSO and RKHS methods resulted in higher genomic heritability for fresh root yield, i.e., 0.679, 0.641, and 0.520, respectively, compared to the other methods (Table 1). However, for dry root yield and dry matter content, the genomic heritability was higher with BayesB and RKHS (0.673 and 0.504 for dry root yield and 0.736 and 0.668 for dry matter content, respectively). Therefore, the BayesB and RKHS methods were able to capture a great part of genomic variation across all traits. In addition, the genomic heritability standard deviation estimates were low, with the exception of the BLASSO method for fresh root yield (0.249) and dry root yield (0.169). In general, the Bayesian methods presented higher standard deviations than the methods predicted by mixed models.

**Table 1:** A posteriori means for genomic estimates of fresh root yield, dry root yield and dry matter content for the first cross-validation strategy.

Methods	${\hat{h}}_{gen}^{2}$	SD ${\hat{h}}_{gen}^{2}$	${\hat{\sigma }}_{g}^{2}$	${\hat{\sigma }}^{2}$	π	λ
	Fresh root yield					
G-BLUP	0.347	0.029	17.01	32.03	-	-
RKHS	0.520	0.026	31.55	29.04	-	-
RR-BLUP	0.376	0.030	19.33	32.04	-	-
BayesB	0.679	0.055	64.82	29.76	0.455	-
BLASSO	0.641	0.249	49.81	28.27	-	140.2
	Dry yield					
G-BLUP	0.332	0.028	1.39	2.81	-	-
RKHS	0.504	0.028	2.61	2.57	-	-
RR-BLUP	0.360	0.029	1.58	2.81	-	-
BayesB	0.673	0.051	5.52	2.62	0.449	-
BLASSO	0.410	0.169	1.67	2.79	-	240.8
	Dry matter content					
G-BLUP	0.517	0.021	2.10	1.96	-	-
RKHS	0.668	0.020	3.58	1.78	-	-
RR-BLUP	0.549	0.021	2.39	1.96	-	-
BayesB	0.736	0.040	5.33	1.87	0.547	-
BLASSO	0.504	0.045	2.13	2.09	-	187.4

$h_{gen}^{2}-$ Genomic heritability; SD $h_{gen}^{2}-$ Genomic heritability standard deviation; ${\hat{\sigma }}_{g}^{2}–$ genotypic variance; ${\hat{\sigma }}^{2}–$ residual variance; π –a priori pi for BayesB method; λ –a priori lambda of BLASSO method.

**Fig 2:**
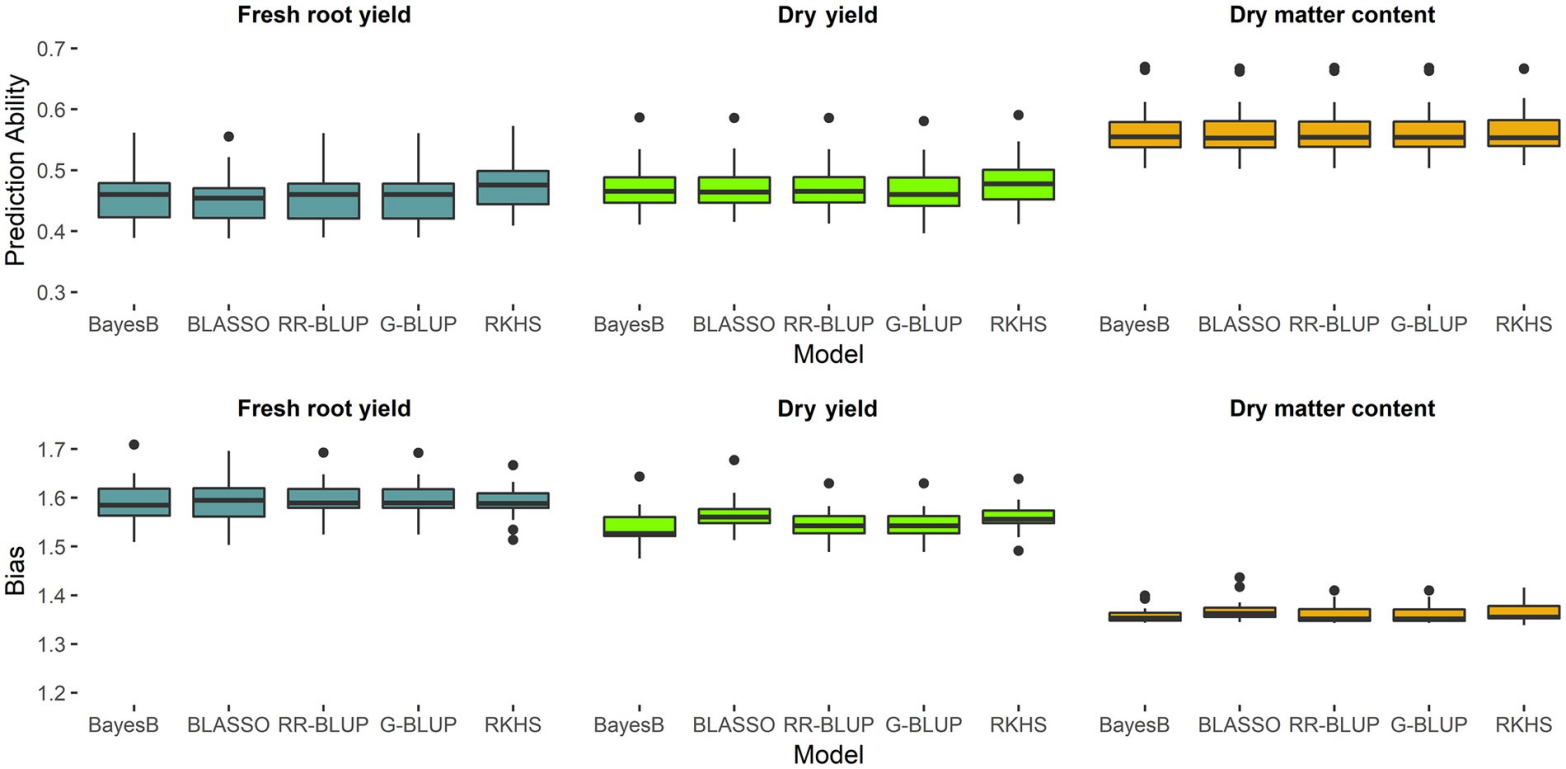
Predictive ability and bias boxplot for different genomic selection methods (BayesB, BLASSO, RR-BLUP, G-BLUP and RKHS) with the 1^st^ cross validation strategy, for fresh root yield, dry root yield and dry matter content.

Similar predictive ability and bias estimates were identified for the different genomic selection methods for the first cross-validation strategy independently of the population structure (Fig 2). On the other hand, regarding the agronomic traits, we observed smaller predictive ability means among the genomic selection methods for fresh root yield (0.4569—RR-BLUP to 0.4756—RKHS) and dry root yield (0.4689—G-BLUP to 0.4818—RKHS) when compared with dry matter content (0.5655—BLASSO to 0.5670—RKHS).

All bias estimates were greater than one, and therefore all genomic selection methods underestimated the marker effects and GEBVs (Fig 2). Among the genomic selection methods, BayesB presented the lowest bias for all agronomic traits, whereas the RKHS method presented lower bias only for fresh root yield (Fig 2). The other genomic selection methods showed similar bias estimates. Comparing traits, dry matter content has smaller bias estimates than fresh root yield and dry root yield, which was expected due to its predominantly additive inheritance and lower environmental influence.

### Deviance analysis

According to the deviance analysis, there were significant differences between the genomic selection methods on the first cross-validation strategy, independently of population structure, especially for fresh root and dry root yield (Table 2). For these two traits, the genomic selection methods had significant differences for predictive ability, while for dry root yield and dry matter content, the genomic selection methods also presented bias estimation differences. Similar results were also observed for prediction accuracies (S3 Table).

**Table 2:** LRT analysis and Tukey’s pairwise test (p≤0.05) for genomic selection prediction for the first cross-validation strategy for fresh root yield, dry root yield and dry matter content.

	DF	Fresh root yield		Dry root yield		Dry matter content	
Deviance		$r_{\hat{y}y}$	*b*	$r_{\hat{y}y}$	*b*	$r_{\hat{y}y}$	*b*
Methods	4	95.62*	1.20	42.44*	60.36*	1.26	23.7*
Cross validation	1	255.41*	50.72*	243.31*	148.42*	271.18*	114.11*
Tukey multiple comparison test							
BayesB		0.4571B (0.0472)	1.5935A (0.0922)	0.4737B (0.0361)	1.5435B (0.0866)	0.5668A (0.0261)	1.3595B (0.0826)
BLASSO		0.4546B (0.0477)	1.5982A (0.0746)	0.4743B (0.0426)	1.5686A (0.0438)	0.5655A (0.0488)	1.3702A (0.0438)
G-BLUP		0.4570B (0.0469)	1.5979A (0.0412)	0.4689C (0.0456)	1.5469B (0.0329)	0.5667A (0.0487)	1.3604B (0.0205)
RKHS		0.4756A (0.0452)	1.5904A (0.0381)	0.4818A (0.0444))	1.5603A (0.0339)	0.5671A (0.0497)	1.3662A (0.0235)
RR-BLUP		0.4570B (0.0411)	1.5980A (0.0929)	0.4740B (0.0329)	1.5469B (0.0870)	0.5666A (0.0205)	1.3605B (0.0811)

$r_{\hat{y}y}$: predictive ability; *b*: bias, DF: degrees of freedom.

*significance deviance for 5% probability in *χ*^2^ test.

Major letters indicate significance differences between genomic prediction methods (p<0.05) by Tukey’s pairwise test. Standard error for the genomic prediction method is in parentheses.

The highest estimates of predictive ability were observed for the RKHS method (p≤0.05) in all traits, except for dry matter content, for which there was no significant difference between the different selection methods (Table 2). The other methods (BayesB, BLASSO, G-BLUP, and RR-BLUP) showed similar predictive abilities for fresh root yield (range of 0.4546 to 0.4571), and the BayesB, BLASSO, and RR-BLUP methods also showed similar results for dry root yield (range of 0.4543 to 0.4737).

Considering the bias, the methods BayesB, G-BLUP and RR-BLUP had the smallest bias estimates for the dry root yield and dry matter content. Therefore, there was no significant difference between the methods for fresh root yield (p≤0.05).

### Correlation of GEBVs among the different genomic selection methods

**Fig 3:**
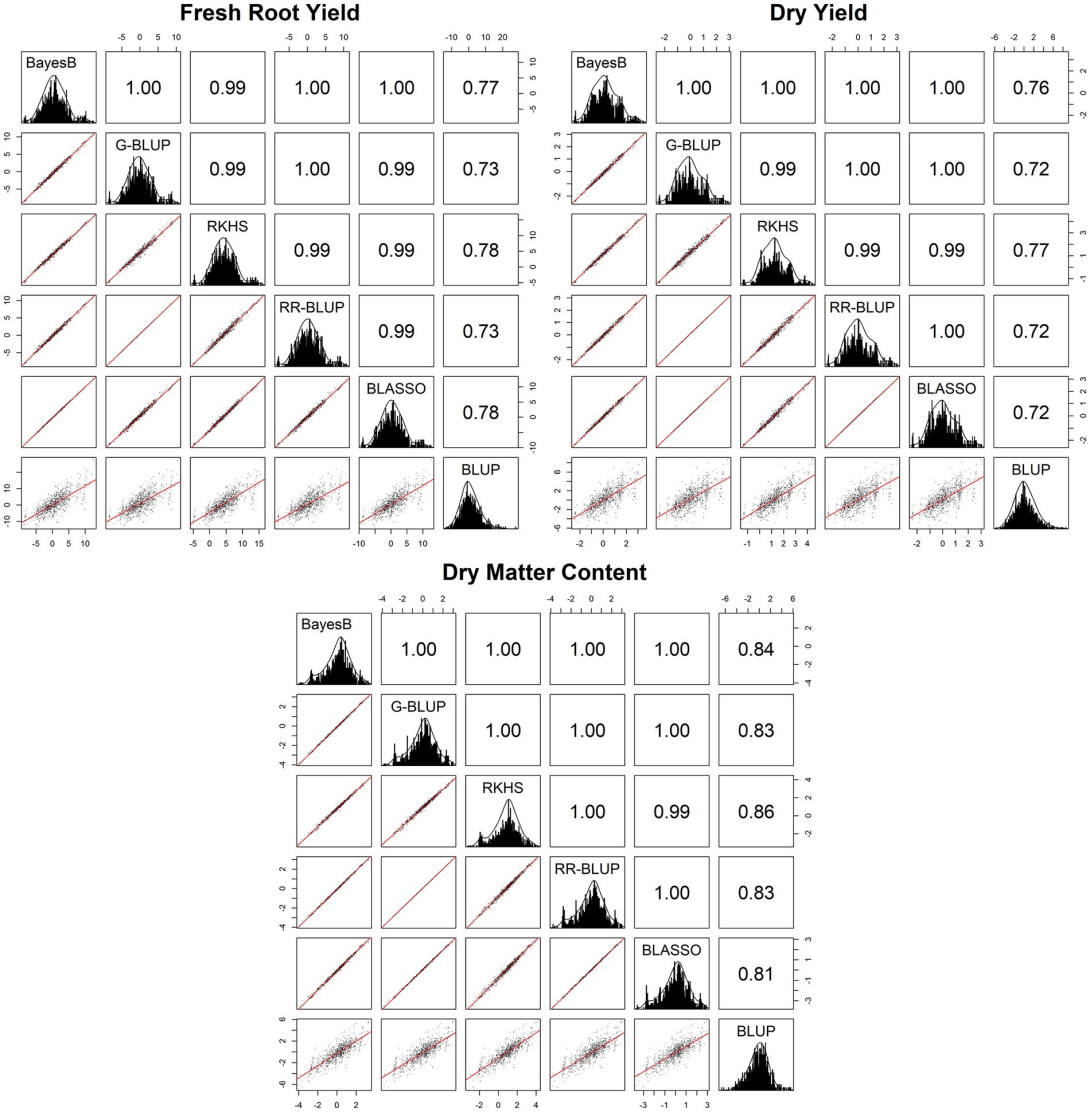
Pearson correlations of genomic estimated breeding values (GEBVs) from different genomic selection methods (BayesB, G-BLUP, RKHS, RR-BLUP and BLASSO) with the 1^st^ cross validation strategy for fresh root yield, dry root yield and dry matter content.

The Pearson correlations of the GEBVs among the genomic selection methods were quite high for dry matter content, fresh root yield and dry root yield, ranging from 0.99 to 1.00 (Fig 3). Therefore, despite some differences in quality prediction parameters, the prediction methods coincided greatly in genotype selection. Therefore, regardless of the genomic selection method applied, the selection for new crosses, the recombination of elite materials or even the clonal selection for new varieties tended to be practically the same in common validation populations. In addition, the correlation of GEBVs for different genomic selection methods with the BLUPs were high for all traits, although the correlations were higher for the dry matter content, whose variation was 0.81 to 0.86.

The RKHS, BLASSO and BayesB methods showed the highest correlations between the genomic selection methods and BLUPs for fresh root yield (0.78, 0.78 and 0.77, respectively), whereas for dry root yield the highest correlations were identified for the RKHS (0.77) and BayesB (0.76) models. Similarly to dry root yield, dry matter content had the highest correlations between GEBVs and BLUPs for the RKHS and BayesB methods, although with higher magnitudes than other traits (0.86 for RKHS and 0.84 for BayesB) (Fig 3).

**Fig 4:**
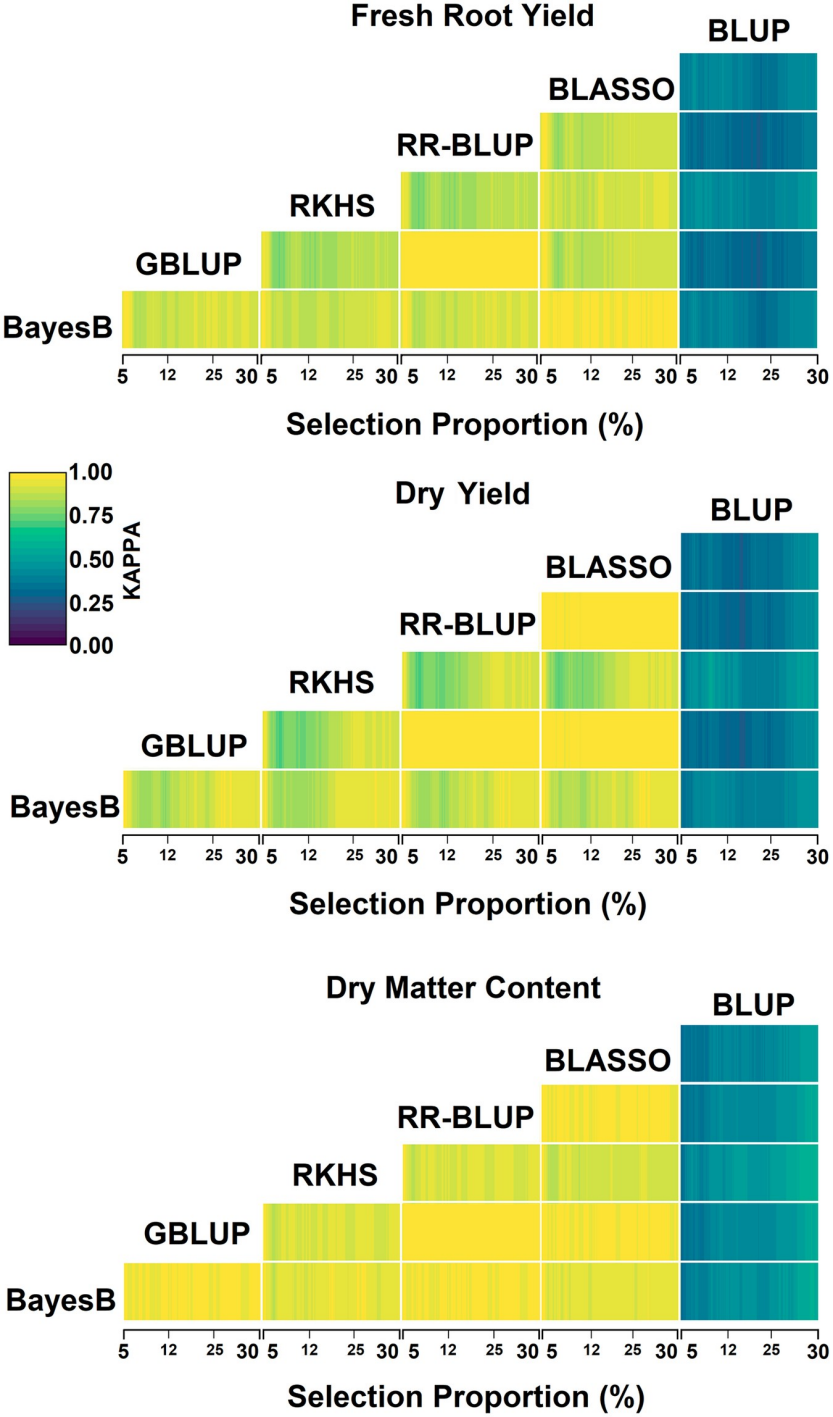
Cohen’s Kappa for the selection coincidence of higher GEBV cassava genotypes, considering selection proportion (5% to 30%—SP) with the 1^st^ cross validation strategy of different genomic selection methods BayesB, BLASSO, G-BLUP, RR-BLUP, RKHS and phenotypic REML/BLUP, for fresh root yield, dry root yield and dry matter content.

The Kappa coefficients of the coincidence selection of the best accessions between different genomic selection methods were high (selection range of 5 to 30%), with a minimum Kappa coefficient of 0.804 considering an 8.1% selection proportion (SP) for fresh root yield (coincidences between the RKHS versus G-BLUP and RR-BLUP methods); 0.761 Kappa at an 8.2% selection proportion (SP) for dry root yield (Kappa between RKHS versus G-BLUP and RR-BLUP); and 0.879 Kappa when selecting 7% of the genotypes for dry matter content (Kappa between RKHS versus G-BLUP and RR-BLUP) (Fig 4). The largest coincidence selection occurred between the G-BLUP and RR-BLUP methods, independent of the SP applied in all evaluated agronomic traits. Therefore, even if there were statistical differences for the predictive ability of the G-BLUP and RR-BLUP methods for dry root yield, the expected genetic gains were practically the same. In addition, the BLASSO method also showed great coincidence in genotype selection with the G-BLUP and RR-BLUP methods for dry root yield and dry matter content, even though there were differences in the GEBV prediction parameters.

The Kappa coefficients among GS methods with BLUPs were high, with the highest estimates closer to 30.0% SP. Additionally, the RKHS method showed less coincidence of selection with the other GS methods but had the highest coincidence with the BLUPs. Thus, it is expected that the RKHS will achieve a greater expected genetic gain, especially for dry root yield, whose coincidence reached 0.634 at 10.4% SP. The RKHS method was also very promising for clone selection of fresh root yield and dry matter content, even with similar results to the other methods. There was a high coincidence of clone selection between the G-BLUP and RR-BLUP. However, G-BLUP had the lowest Kappa coefficients with BLUPs (0.405 for 16% SP), resulting in the lowest expected genetic gain.

### Training population clustering

**Fig 5:**
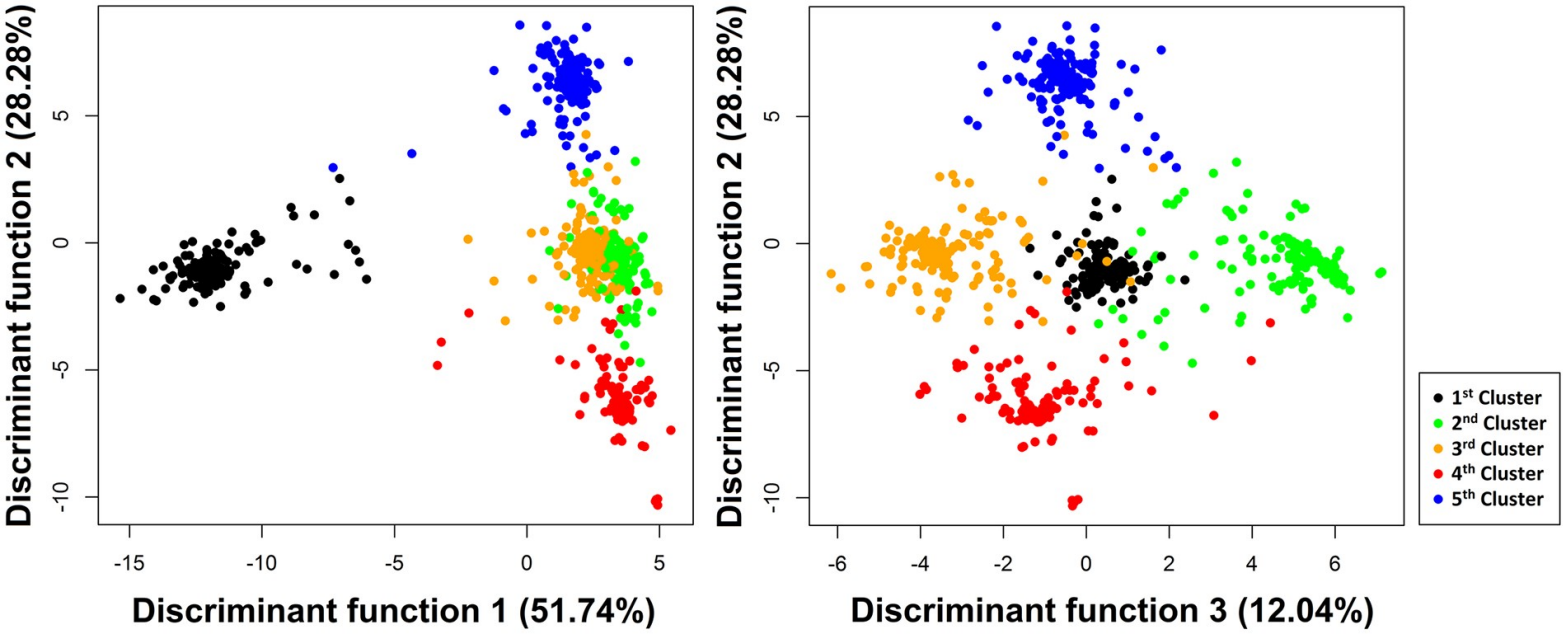
Distribution of the five clusters identified by discriminant analysis of principal components of single nucleotide polymorphism markers (SNPs).

The DAPC grouped the cassava accessions into five clusters based on the genomic relationship matrix (Fig 5). The distribution of the accessions into different clusters was quite balanced, with 162, 175, 185, 155 and 211 accessions present in clusters 1, 2, 3, 4, and 5, respectively. The first three discriminant functions were able to capture most of the genomic data variation, explaining more than 92% of the variance. Therefore, these functions might represent the relationship of the cassava accessions in each cluster with high efficiency (Fig 5). Considering the 1^st^ and 2^nd^ discriminant functions, cluster 1 was the most distinct, while clusters 2, 3, 4, and 5 had closer genetic relationships. Based on the 1^st^ and 2^nd^ discriminant functions, there was an overlap of the cassava accessions belonging to clusters 2 and 3 due to the stronger relationship between the clusters. For the 2^nd^ and 3^rd^ discriminant functions, the clusters were distributed in the four quadrants with overlaps of accessions between clusters. The accessions from cluster 1 overlapped with some accessions from clusters 2 and 3, also indicating some genetic relationship between these clusters. We also found a low correlation between the cyanide content of the germplasm and the DAPC clustering, as four of the five clusters were grouped with sweet and bitter cassava. Only cluster 1 was composed primarily of bitter cassava.

### Efficiency and consistency of genomic selection methods in different population structure scenarios

The population structure estimated based on the DAPC was used to evaluate the predictions of the different genomic selection methods, considering modified cross validation between and within clusters. The results presented in this section were obtained using the 2^nd^, 3^rd^, and 4^th^ cross validation strategies. According to the deviance analysis of between- and within-cluster validations, the population structure and cross validation factors were significant for all traits. However, after including the population structure of the accessions, there was a reduction in the predictive ability of the genomic prediction models, since the variance of the population structure was removed from cross validation variance (Tables 2 and 3). The prediction accuracy had high correlation with predictive ability for all the genomic selection methods (S4 Table). This happened due prediction accuracy is estimate by predictive ability divided by the square root of the phenotypic trait heritability, which is a constant.

**Table 3:** LRT analysis and Tukey’s pairwise test (p≤0.05) for genomic selection prediction considering the 2^nd^, 3^rd^, and 4^th^ cross-validation strategies to estimate the effect of population structure for fresh root yield, dry root yield and dry matter content.

Deviance	DF	Fresh root yield		Dry root yield		Dry matter content	
		$r_{\hat{y}y}$	*b*	$r_{\hat{y}y}$	*b*	$r_{\hat{y}y}$	*b*
Methods	4	79.75*	855.48*	70.99*	479.64*	10.81*	375.23*
Cross validation	1	581.54*	85.18*	512.58*	149.22*	490.46*	277.74*
Population structure	1	1956.77*	342.7*	1716.17*	611.94*	1466.70*	778.03*
Tukey multiple comparison test							
BayesB		0.0821B (0.1770)	1.5013B (0.0902)	0.1327B (0.1539)	1.4778C (0.0805)	0.2675B (0.1480)	1.3458C (0.0818)
BLASSO		0.0773B (0.1738)	1.4105C (0.1059)	0.1434B (0.1604)	1.6179B (0.1330)	0.2779AB (0.1382)	1.4896A (0.1461)
G-BLUP		0.0848B (0.1756)	1.6941A (0.1856)	0.1415B (0.1808)	1.6519A (0.0795)	0.2735AB (0.1427)	1.4179B (0.1397)
RKHS		0.1176A (0.1680)	1.6866A (0.1995)	0.1722A (0.1808)	1.6631A (0.0795)	0.2840A (0.1366)	1.4181B (0.1443)
RR-BLUP		0.0858B (0.1736)	1.6903A (0.1870)	0.1415B (0.1808)	1.6493A (0.0795)	0.2752AB (0.1459)	1.4214B (0.1414)

$r_{\hat{y}y}$: predictive ability; *b*: bias, DF: degrees of freedom.

*significance deviance for 5% probability in *χ*^2^ test.

Major letters indicate significance difference between genomic prediction methods (p<0.05) by Tukey’s pairwise test. Standard error for the genomic prediction method is in parentheses.

When the population structure was included in the predictions, the factor of genomic selection method showed significance of predictive ability for all traits. Similar to the estimates obtained without population structure control, the RKHS method yielded higher predictive ability in comparison to the other genomic selection methods. However, for dry matter content, the predictive ability of RKHS was only higher than that of BayesB (p≤0.05) (Table 3). The RKHS method was also highlighted by its greater consistency between the different population structure scenarios (S4, S5 and S6 Figs). This may be because the Gaussian kernel matrix predicts not only additive effects but also non-additive effects, which is the unique contrasting assumption with the G-BLUP method.

The predictive ability estimates for all traits had low consistency between different population structure scenarios (S4, S5 and S6 Figs) and small differences between genomic selection methods in within-cluster cross validations (S4A, S5A and S6A Figs). In the cross validation between clusters, the predictive ability differences for genomic selection methods were also small, although the RKHS method presented higher predictive ability for the fresh and dry root yield traits between the different scenarios (S4C, S5C and S6C Figs). The predictions of the cross validations between clusters composed of three or four DAPC clusters in the training population were similar among the genomic selection methods, although of smaller magnitude in relation to those found in the cross validation without the population structure control. Therefore, the population structure had a great effect on the genomic prediction efficiency, even presenting a predictive ability close to zero in some situations for yield traits.

The cross validations between four clusters composing the training population present smaller but consistent predictive ability estimates than those cross validations formed by three clusters.

The RKHS method had the highest estimate of predictive ability for all traits (p≤0.05) (Table 3), but also was the most biased method. In general, Bayesian methods were less biased than REML/BLUP methods for all traits (Table 3). In addition, the between-clusters cross validations with four clusters as the training population showed smaller bias estimates, with emphasis on the RKHS method (S7, S8 and S9 Figs).

The fresh root yield and dry root yield had similar results under different genomic prediction scenarios. For these two traits, it was observed that the absence of cluster 2 from the training population increased the prediction efficiency of the other clusters in all of the prediction scenarios, whereas the absence of cluster 5 from the training population reduced the prediction efficiency for all validation clusters except for cluster 1. In the validation of cluster 4, the absence of cluster 1 dramatically reduced the predictive ability in comparison with cluster 5. In addition, the absence of cluster 3 reduced the genomic selection efficiency when validating cluster 5.

For dry matter content, the presence of cluster 5 in the training populations was important only for clusters 1 and 2 prediction (S5 Table; S6 and S9 Figs), as its absence decreased the predictive ability estimates for these clusters. The absence of cluster 2 from the training populations reduced predictive ability to almost zero when validation was performed for cluster 5. Moreover, the presence of cluster 3 in the training populations was more important for cluster 4 prediction. On the other hand, the validation of cluster 3 did not depend on the presence of any DAPC cluster in the training population, since all estimates of genomic selection efficiency were similar in the different cross validation scenarios between clusters. Thus, the higher the genetic relationship between training and validation populations, the higher the genomic selection efficiency, possibly due to the weaker effect of population structure on prediction estimates.

The RKHS method showed higher efficiency and consistency in most validation scenarios, even in between-cluster cross validation scenarios with three or four clusters as the training population. Therefore, RKHS results in higher genetic gain for clonal selection in cassava for yield traits.

## Discussion

### Phenotypic versus genomic heritability and its implications for genomic selection in cassava

Fresh root yield, dry root yield and dry matter content are important traits in cassava breeding for industrial uses. However, the phenotype heritability estimates in this work were extremely influenced by the environment, considering their low magnitudes (0.337, 0357, and 0.545 for fresh root yield, dry root yield and dry matter content, respectively). In addition, fresh and dry root yields do not have a high correlation between the seedling phase and the clone performance in the advanced stages of the cassava breeding program due to morphological differences between the roots of seedling nursery plants and those of stem cutting plants [6]. Therefore, considering these factors and the low heritability of these traits, clone and parental phenotypic selection at the seedling phase tends to be ineffective.

Previous studies of general and specific combining abilities in cassava have shown that fresh root yield, number of roots, harvest index and plant height traits have a predominant non-additive effect, while dry matter content and root diameter are predominantly governed by additive effect [47–49]. Thus, the dry matter content is a trait that allows greater predictive ability, even in earlier breeding program stages, whereas parental selection for yield traits is more complex, requiring more refined strategies for analysis and selection [49].

The genomic heritability indicates the adjustment of marker distribution effects on trait heredity and variation. The genomic selection methods evaluated gave higher heritability estimates than the phenotypic heritability. This implied that all the genomic methods overestimated the marker effects except G-BLUP, which was consistent with the phenotypic heritability for fresh and dry root yields. Methodologically, the RKHS and BayesB methods presented a better adjustment to the genetic variation of cassava agronomic traits. However, the G-BLUP method presented the closest genomic heritability estimates to the phenotypic heritability found in advanced field trials in cassava breeding programs.

### Genomic selection efficiency considering different prediction models

Although the differences between the genomic selection methods were not of high magnitude for predictive ability, in the cross validation scenario independent of the population structure (1^st^ cross-validation strategy), the RKHS method had slightly higher predictive ability compared to the other genomic selection methods for fresh root yield (0.4756) and dry root yield (0.4818). Wolfe et al. [4] also reported lower predictive ability for fresh root yield (0.18 to 0.37) and dry matter content (0.24 to 0.68) and did not find great differences between the genomic selection methods, especially for dry matter content. Although Wolfe et al. [4] explored different methods to evaluate the dry matter content (specific gravity and oven method), our predictive ability results were higher than theirs estimated by the gravity method. Moreover, according to these authors, the RKHS method was more accurate for the following traits: fresh root yield (0.21 to 0.37), harvest index (0.41 to 0.48), number of roots (0.25 to 0.39), shoot yield (0.24 to 0.39), and initial vigor (0.18 to 0.38), the amplitude being due to the different breeding programs evaluated. Small differences between genomic selection methods have also been reported for Cassava Brown Streak Disease (CBSD) virus resistance, with a predictive ability of 0.27 to 0.42 [50]. Several other authors have also evaluated different genomic selection methods and reported small differences between methodologies in both simulated data [7, 14] as well as real data for maize, wheat and barley [19, 42, 51].

In general, genomic selection methods with the same genetic assumptions tend to have similar predictive ability. However, the inclusion of an extra genetic effect in the predictions can result in differences in predictive ability, even using the same prediction method [26], which in fact was found in the present work for fresh root yield due to the great influence of non-additive effects.

The RKHS method was more promising for clone selection, despite the genetic relationship between the training and validation populations, because it presented a good adjustment for prediction of cassava agronomic traits. The RKHS method has similar assumptions to G-BLUP, except for the genetic relationship matrix. So, it is possible that the method’s superiority is related to prediction of partial non-additive plus additive effects [52–53].

The nonlinear models such as RKHS allow the estimation of additional genetic variance fractions, since the inheritance and variation of complex traits are nonlinear [52]. Morota and Gianola [53] reported that semi-parametric methods have great potential to estimate all additive and non-additive effects in real data. In clonal propagated cultures such as cassava, non-additive effects may have an important influence on phenotype expression and clone selection [26]. These methods therefore, are of interest in cassava breeding since additive and dominant genetic effects explain much of cassava’s genetic variation [5]. In this case, methods that can predict the non-additive genetic effects are quite useful at early stages in clone selection aimed at the development of new varieties. Indeed, the RKHS method was recommended by Heslot et al. [19] for clone selection due to its high predictive ability and good fit, even if it was more biased than other genomic selection methods.

### Population structure effect in genomic prediction

The population structure effect, evaluated using by the DAPC method, was quite pronounced in different cross validation scenarios for genomic prediction efficiency estimates (2^nd^, 3^rd^, and 4^th^ cross validation strategies). In general, the estimates of predictive ability and bias were high for cross validation with total accession randomization, followed by within-cluster cross validation and lastly by between-clusters cross validation. The within-cluster cross validation did not outperform the all randomized cross validation, as expected in genomic selection for inbred populations with smaller effective population size [13,54–57]. This may be related to the training population’s lack of kinship relationship due to having been formed by germplasm bank accessions with high heterozygosity loci in each cassava clone.

Wolfe et al. [4] evaluated the possibility of using a training population composed of data from different breeding programs in order to increase the predictive ability. These authors reported that even in highly related populations, this approach was only effective when some of the validation population clones were in the training population. Ly et al. [25] have also shown that higher estimates of predictive ability are associated with higher levels of genetic relationship in the population. Therefore, the higher the genetic relationship between individuals, the greater the haplotype blocks in linkage disequilibrium (LD), which increases the probability that at least one marker is in linkage disequilibrium (LD) with a QTL. Slatkin [58] reported that one marker in total linkage disequilibrium (LD) with each haplotype block is enough to identify the association between the molecular marker and the trait under analysis. According to Sorkheh et al. [59], the factors that contribute to increased LD are high inbreeding rates, a low recombination rate, small population size, genetic drift, migration, morphology or geographical isolation and epistasis.

In order to maintain the predictive ability of genomic selection between generations, it is recommended to conduct recurrent selection [4], with a large number of progeny to maintain the effective population size. Currently there are training optimization algorithms such as the STPGA [60], when allied with recurrent selection, promises to increase the genomic selection efficiency. This algorithm selects individuals to represent the prediction population to be phenotyped, aiming only to increase or maintain the efficiency of genomic prediction for the next generations [4]. Therefore, ignoring the population structure in genomic selection may compromise the methodology’s efficiency and expected results.

### Potential application of genomic selection in cassava

Considering that inbreeding depression is quite pronounced for cassava yield traits such as fresh root yield (range of 1.78% to 55.20%, mean 19.38%) and dry root yield (range of 0% to 55.57%, mean 17.54%) [6,61], most cassava breeding programs use heterozygous progenitors to generate highly segregant F_1_ populations [3], and therefore, each F_1_ individual is a single recombinant event. In general, this hinders the selective process in the early stages of breeding, since heritability estimates in the seedling stage are quite biased. On the other hand, the clonal reproduction of cassava seedlings allows the exponential multiplication of individuals, allowing the estimation of all genetic variation and the genetic effects inheritance in controlled trials.

Due to the advantages of clonal propagation, the family structure is usually neglected by breeders in clonal selection [5], with little adoption of pedigree information. Thus, genomic selection methods using a genetic relationship matrix, such as G-BLUP and RKHS, may contribute to increasing selection gains, using covariance information among individuals for estimation of GEBVs. These methodologies are more efficient than pedigree prediction since they estimate relationships by markers, even without previous kinship knowledge [39]. According to Edriss et al. [62], the G-BLUP method is greatly superior to the pedigree prediction method, with a predictive ability nine times higher than that provided by the pedigree matrix.

Genomic selection has been used as an important method for selection of complex traits controlled by several small effects QTLs [23]. According to Crossa et al. [9], the results of genomic selection have been quite promising when applied in breeding program phases or situations in which phenotypic selection is impossible or inefficient. Even with low predictive ability, genomic selection might allow shortening of the recurrent selection cycle [8] or the discarding of undesirable individuals, thereby reducing phenotyping costs [62], which in most cases are high. An example is the work of Wolfe et al. [27], who conducted two recurrent selection cycles over two years, increasing the cassava African mosaic virus resistance allelic frequency from 44% to 63%. This selection gain evidences the potential of genomic selection in the early stages (mainly seedlings), resulting in shortening of the breeding program selection cycle. Using conventional methods, it would take at least eight years in total to complete two cycles of phenotypic selection.

The success of genomic selection for African cassava mosaic virus resistance [27] is due to the high genomic heritability of this trait (56.5%) [63]. However, for complex traits such as fresh and dry root yields, the inclusion of non-additive effects in the prediction model tends to increase genomic selection efficiency, since the non-additive genetic variation prevails for low-heritability cassava traits. Thus, genomic selection methods with non-additive effects prediction should be considered [26–27].

## Supporting information

S1 Fig Heat map of pairwise linkage disequilibrium measurements for the single nucleotide polymorphism markers in 888 cassava accessions. (TIF)

S2 Fig Cumulative variance of the principal components estimated from additive relationship matrix (G). (TIF)

S3 Fig Variance of the first four discriminant functions of principal components estimated from additive relationship matrix (G), and clustering of the discriminant analysis of principal components. (TIF)

S4 Fig Predictive ability boxplots of different genomic selection methods for fresh root yield for 2^nd^, 3^rd^, and 4^th^ cross-validation strategies. (**A**): Validation and training within clusters created by Discriminant Analysis of Principal Components (DAPC), informed in line. **(B)**: Validation in DAPC line cluster and training population with all the remaining DAPC clusters. **(C)**: Validation in DAPC line cluster and training with DAPC clusters column informed. Colors represent the absent cluster in training population. Black– 1^st^ Cluster; Green– 2^nd^ Cluster; Orange– 3^rd^ Cluster; Red– 4^th^ Cluster; Blue– 5^th^ Cluster; Brown–None absent Cluster.
(TIF)

S5 Fig Predictive ability boxplots of different genomic selection methods for dry yield for 2^nd^, 3^rd^, and 4^th^ cross-validation strategies. (**A**): Validation and training within clusters created by Discriminant Analysis of Principal Components (DAPC), informed in line. **(B)**: Validation in DAPC line cluster and training population with all the remaining DAPC clusters. **(C)**: Validation in DAPC line cluster and training with DAPC clusters column informed. Colors represent the absent cluster in training population. Black– 1^st^ Cluster; Green– 2^nd^ Cluster; Orange– 3^rd^ Cluster; Red– 4^th^ Cluster; Blue– 5^th^ Cluster; Brown–None absent Cluster.
(TIF)

S6 Fig Predictive ability boxplots of different genomic selection methods for dry matter content for 2^nd^, 3^rd^, and 4^th^ cross-validation strategies. (**A**): Validation and training within clusters created by Discriminant Analysis of Principal Components (DAPC), informed in line. **(B)**: Validation in DAPC line cluster and training population with all the remaining DAPC clusters. **(C)**: Validation in DAPC line cluster and training with DAPC clusters column informed. Colors represent the absent cluster in training population. Black– 1^st^ Cluster; Green– 2^nd^ Cluster; Orange– 3^rd^ Cluster; Red– 4^th^ Cluster; Blue– 5^th^ Cluster; Brown–None absent Cluster.
(TIF)

S7 Fig Bias boxplots of different genomic selection methods for fresh root yield for 2^nd^, 3^rd^, and 4^th^ cross-validation strategies. (**A**): Validation and training within clusters created by Discriminant Analysis of Principal Components (DAPC), informed in line. **(B)**: Validation in DAPC line cluster and training population with all the remaining DAPC clusters. **(C)**: Validation in DAPC line cluster and training with DAPC clusters column informed. Colors represent the absent cluster in training population. Black– 1^st^ Cluster; Green– 2^nd^ Cluster; Orange– 3^rd^ Cluster; Red– 4^th^ Cluster; Blue– 5^th^ Cluster; Brown–None absent Cluster.
(TIF)

S8 Fig Bias boxplots of different genomic selection methods for dry yield for 2^nd^, 3^rd^, and 4^th^ cross-validation strategies. (**A**): Validation and training within clusters created by Discriminant Analysis of Principal Components (DAPC), informed in line. **(B)**: Validation in DAPC line cluster and training population with all the remaining DAPC clusters. **(C)**: Validation in DAPC line cluster and training with DAPC clusters column informed. Colors represent the absent cluster in training population. Black– 1^st^ Cluster; Green– 2^nd^ Cluster; Orange– 3^rd^ Cluster; Red– 4^th^ Cluster; Blue– 5^th^ Cluster; Brown–None absent Cluster.
(TIF)

S9 Fig Bias boxplots of different genomic selection methods for dry matter content for 2^nd^, 3^rd^, and 4^th^ cross-validation strategies. (**A**): Validation and training within clusters created by Discriminant Analysis of Principal Components (DAPC), informed in line. **(B)**: Validation in DAPC line cluster and training population with all the remaining DAPC clusters. **(C)**: Validation in DAPC line cluster and training with DAPC clusters column informed. Colors represent the absent cluster in training population. Black– 1^st^ Cluster; Green– 2^nd^ Cluster; Orange– 3^rd^ Cluster; Red– 4^th^ Cluster; Blue– 5^th^ Cluster; Brown–None absent Cluster.
(TIF)

S1 Table Multilocus heterozygosity of individual level. (XLSX)

S2 Table Heterozygosity of SNP markers. (XLSX)

S3 Table LRT analysis and Tukey’s pairwise test (p≤0.05) for prediction accuracies for the first cross validation strategy for fresh root yield, dry root yield and dry matter content. (DOCX)

S4 Table LRT analysis and Tukey’s pairwise test (p≤0.05) of the prediction accuracies considering the 2^nd^, 3^rd^, and 4^th^ cross validation strategies to estimate the effect of population structure for fresh root yield, dry root yield and dry matter content. (DOCX)

S5 Table Different genomic selection Best linear unbiased prediction (BLUPs) scenarios due Discriminant Analysis of Principal Components (DAPC) cassava accessions clustering for fresh root yield, dry root yield and dry matter content. (DOCX)
