# Supplementary figures and images for: Cassava yield traits predicted by genomic selection methods
Source: PLoS One. 2019 Nov 14;14(11):e0224920. doi: 10.1371/journal.pone.0224920 (PMC6855463; doi:10.1371/journal.pone.0224920)

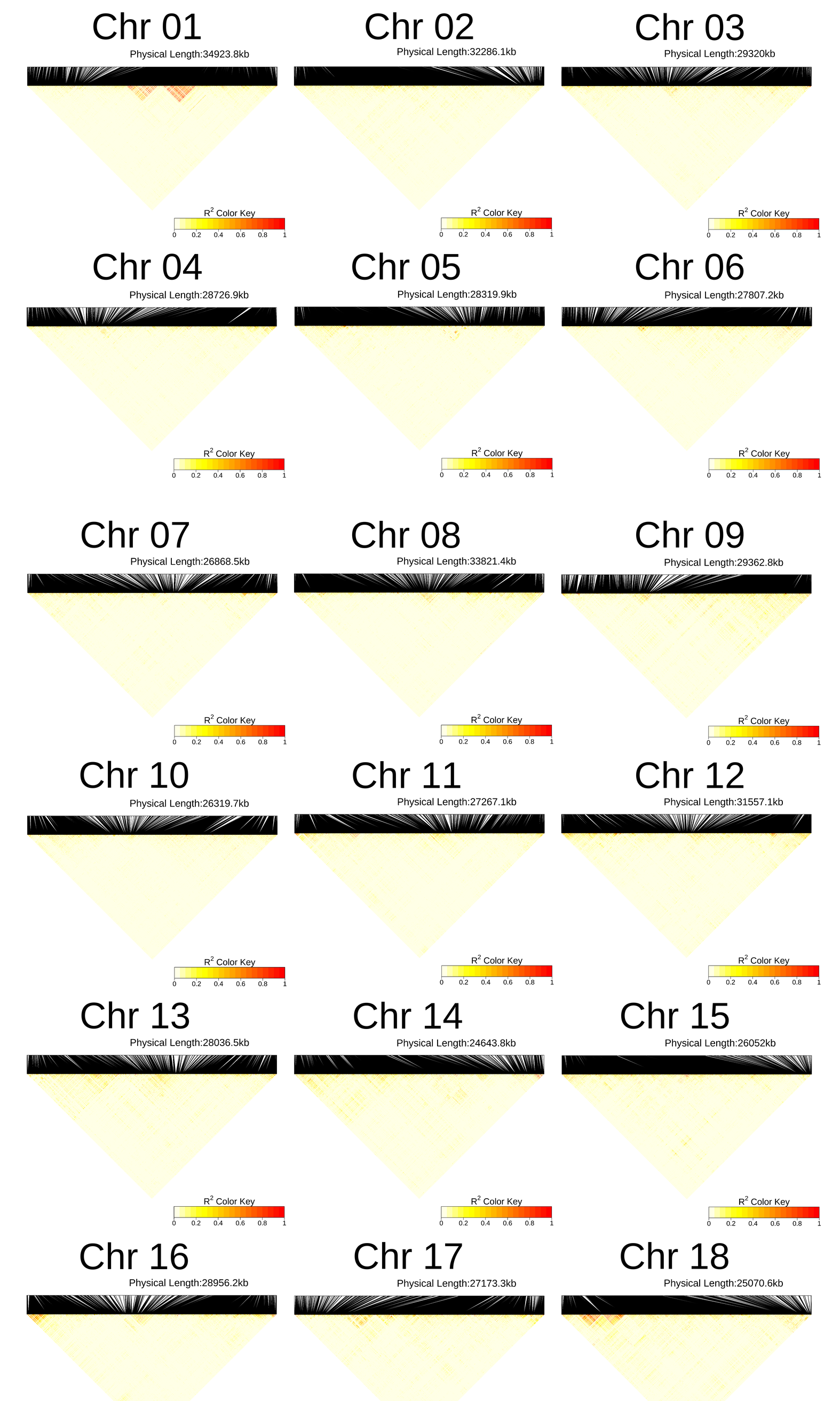

Supplement: S1 Fig — (TIF) [file pone.0224920.s001.tif]

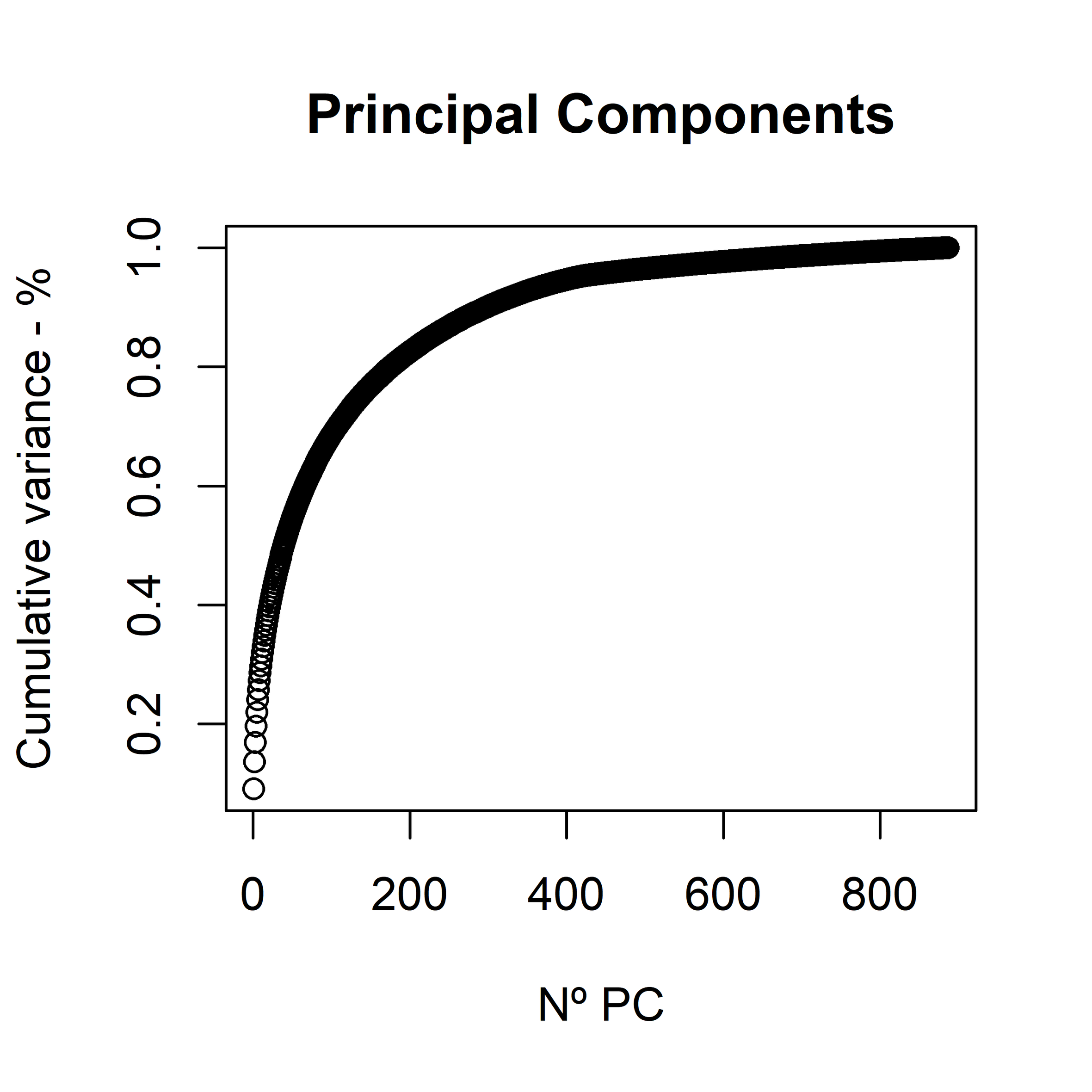

Supplement: S2 Fig — (TIF) [file pone.0224920.s002.tif]

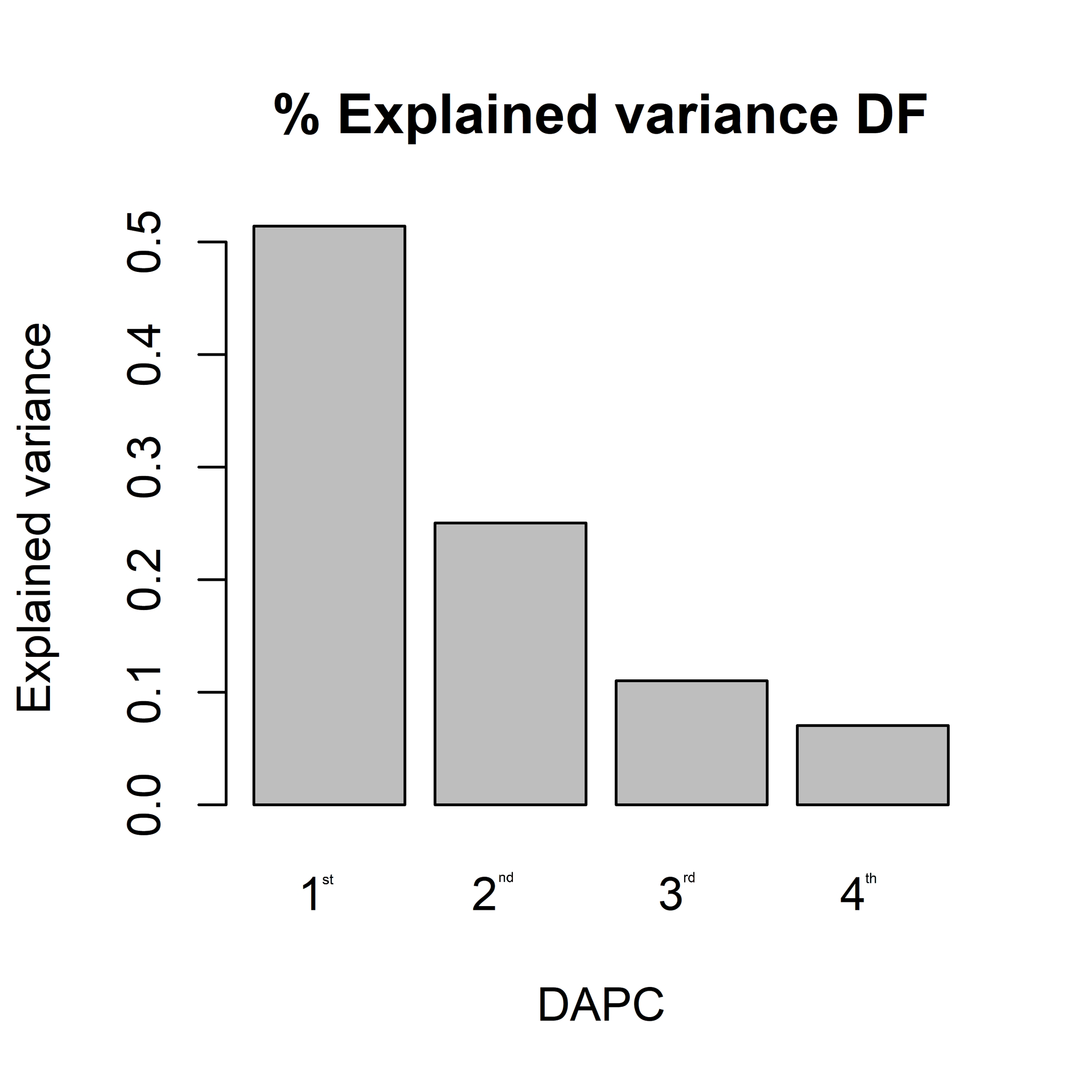

Supplement: S3 Fig — (TIF) [file pone.0224920.s003.tif]

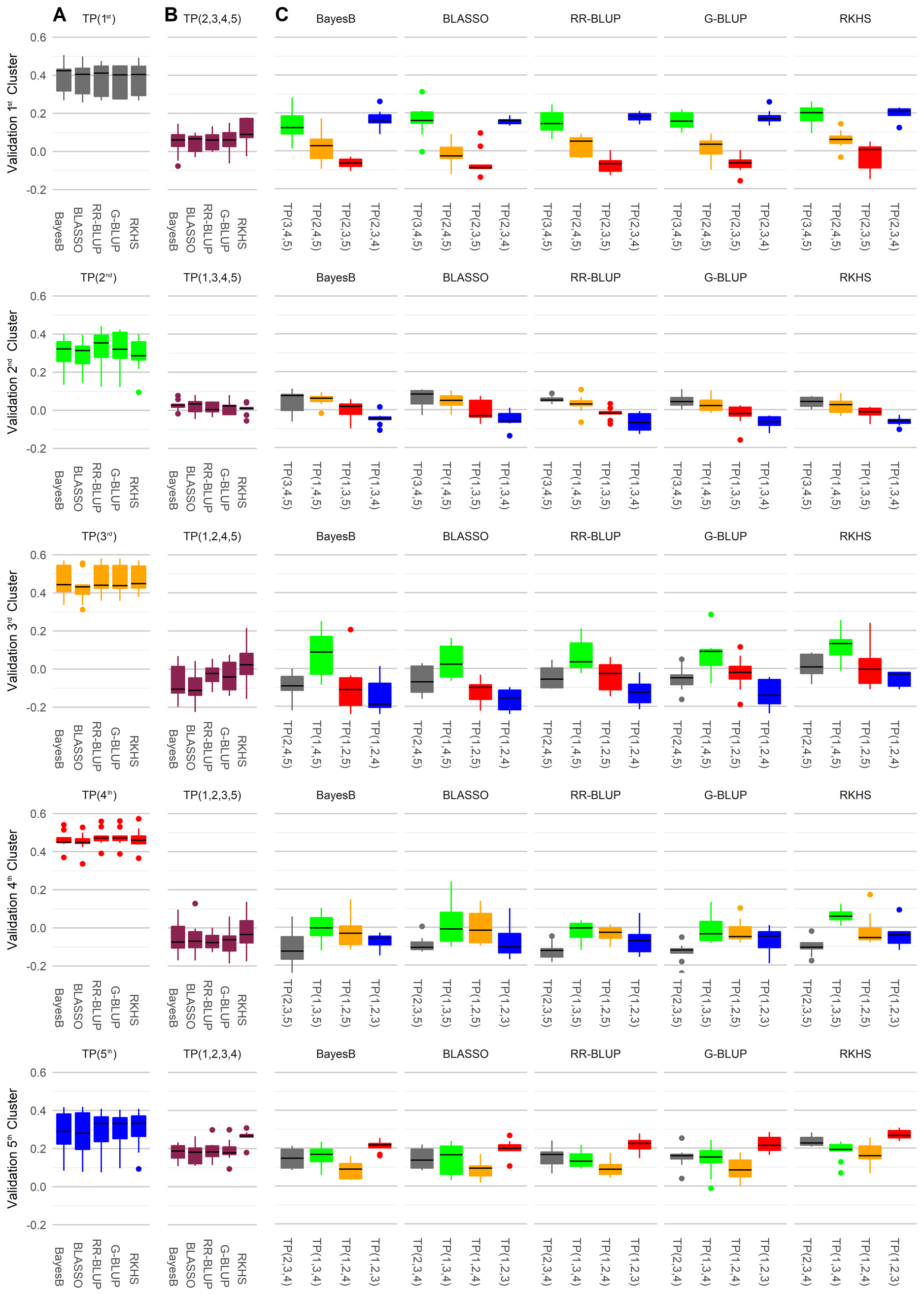

Supplement: S4 Fig — (A): Validation and training within clusters created by Discriminant Analysis of Principal Components (DAPC), informed in line. (B): Validation in DAPC line cluster and training population with all the remaining DAPC clusters. (C): Validation in DAPC line cluster and training with DAPC clusters column informed. Colors represent the absent cluster in training population. Black– 1st Cluster; Green– 2nd Cluster; Orange– 3rd Cluster; Red– 4th Cluster; Blue– 5th Cluster; Brown–None absent Cluster. (TIF) [file pone.0224920.s004.tif]

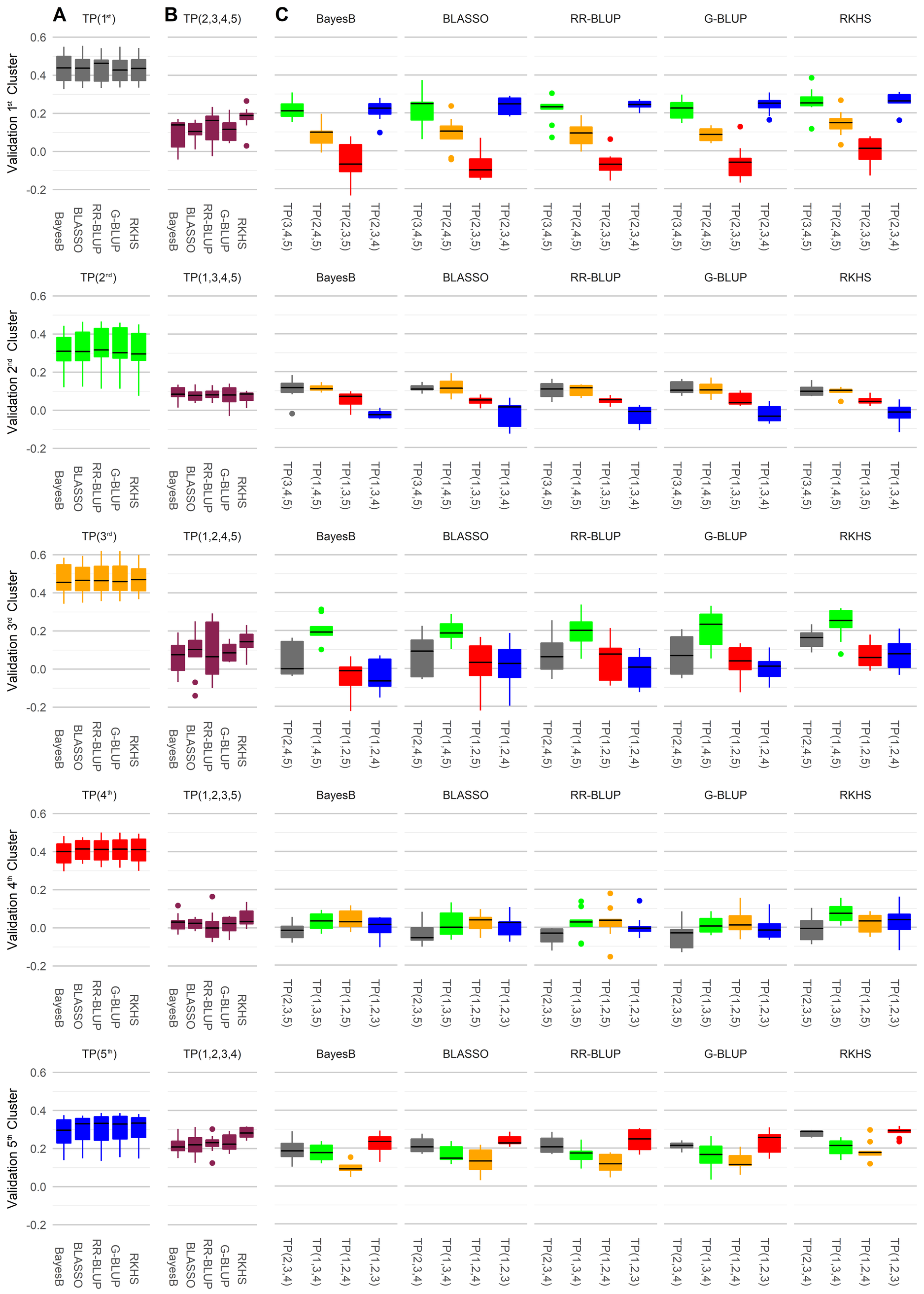

Supplement: S5 Fig — (A): Validation and training within clusters created by Discriminant Analysis of Principal Components (DAPC), informed in line. (B): Validation in DAPC line cluster and training population with all the remaining DAPC clusters. (C): Validation in DAPC line cluster and training with DAPC clusters column informed. Colors represent the absent cluster in training population. Black– 1st Cluster; Green– 2nd Cluster; Orange– 3rd Cluster; Red– 4th Cluster; Blue– 5th Cluster; Brown–None absent Cluster. (TIF) [file pone.0224920.s005.tif]

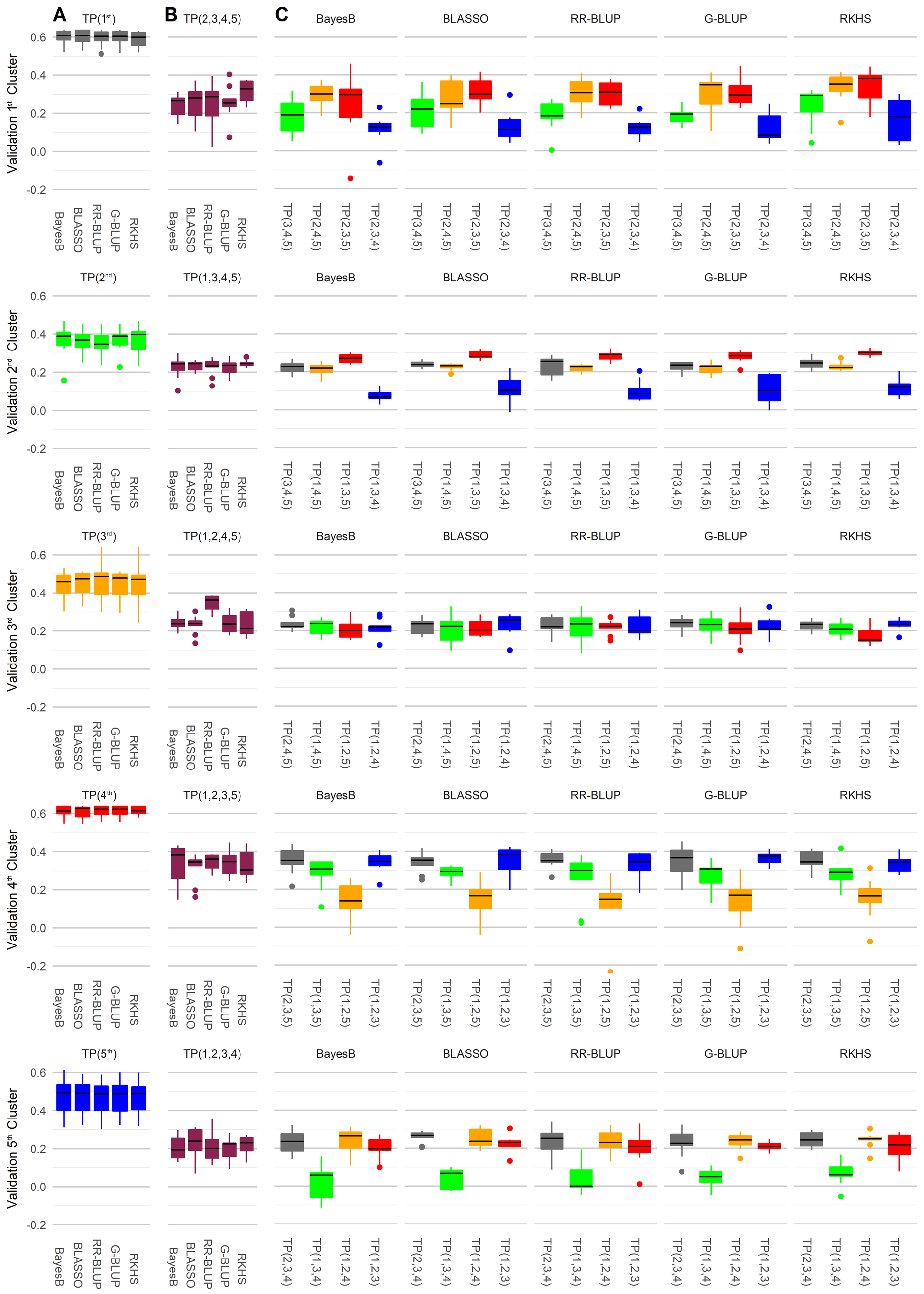

Supplement: S6 Fig — (A): Validation and training within clusters created by Discriminant Analysis of Principal Components (DAPC), informed in line. (B): Validation in DAPC line cluster and training population with all the remaining DAPC clusters. (C): Validation in DAPC line cluster and training with DAPC clusters column informed. Colors represent the absent cluster in training population. Black– 1st Cluster; Green– 2nd Cluster; Orange– 3rd Cluster; Red– 4th Cluster; Blue– 5th Cluster; Brown–None absent Cluster. (TIF) [file pone.0224920.s006.tif]

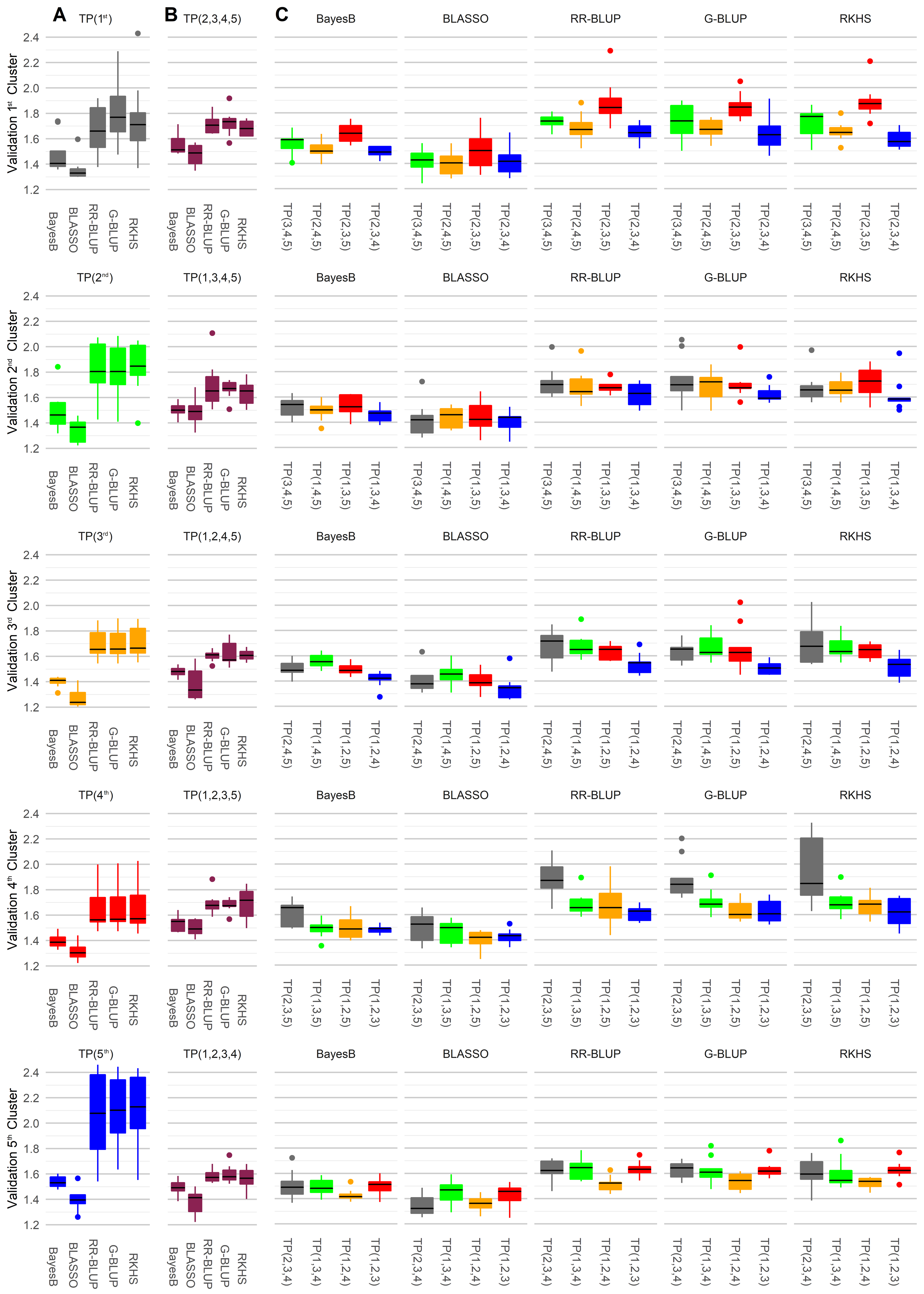

Supplement: S7 Fig — (A): Validation and training within clusters created by Discriminant Analysis of Principal Components (DAPC), informed in line. (B): Validation in DAPC line cluster and training population with all the remaining DAPC clusters. (C): Validation in DAPC line cluster and training with DAPC clusters column informed. Colors represent the absent cluster in training population. Black– 1st Cluster; Green– 2nd Cluster; Orange– 3rd Cluster; Red– 4th Cluster; Blue– 5th Cluster; Brown–None absent Cluster. (TIF) [file pone.0224920.s007.tif]

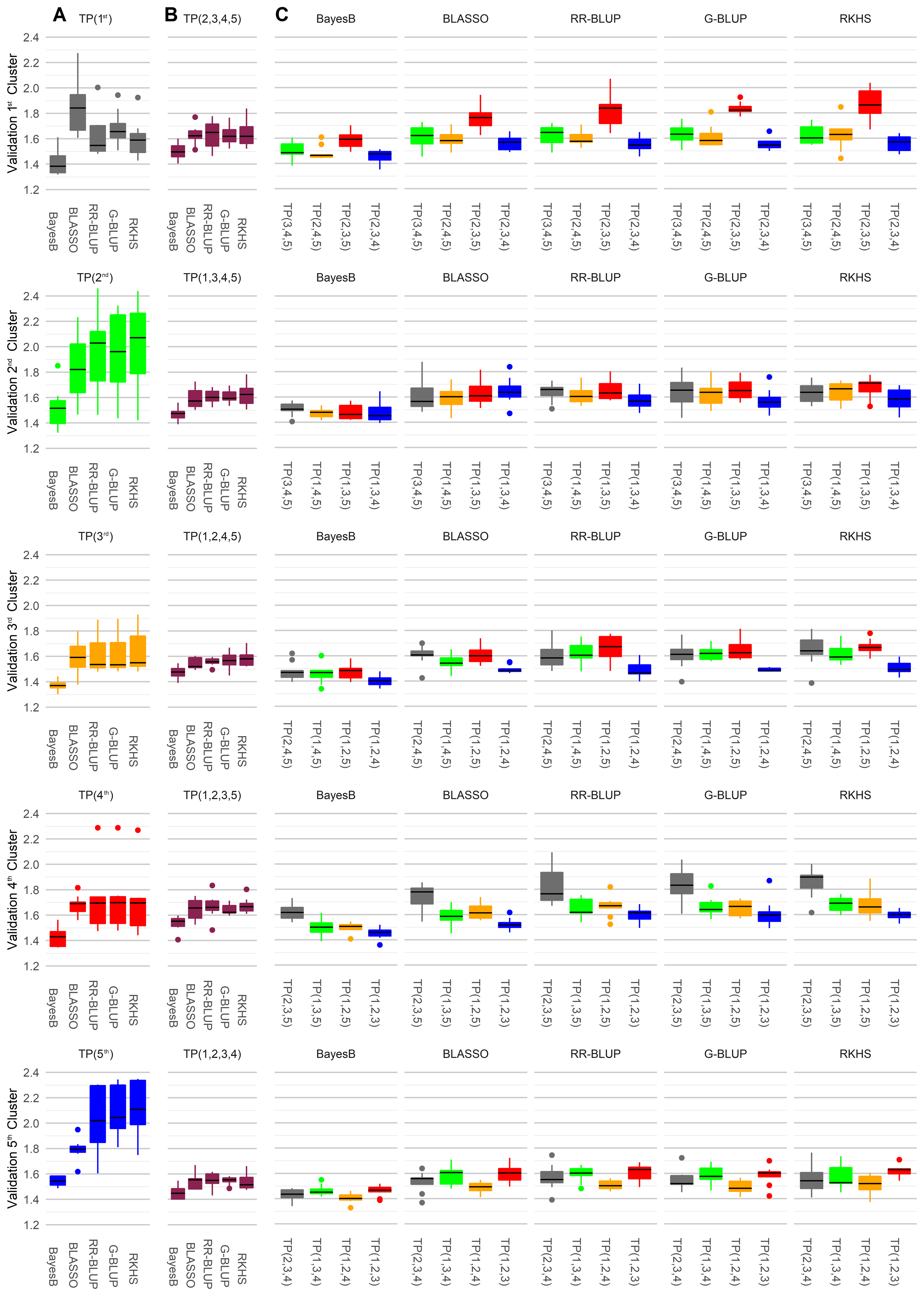

Supplement: S8 Fig — (A): Validation and training within clusters created by Discriminant Analysis of Principal Components (DAPC), informed in line. (B): Validation in DAPC line cluster and training population with all the remaining DAPC clusters. (C): Validation in DAPC line cluster and training with DAPC clusters column informed. Colors represent the absent cluster in training population. Black– 1st Cluster; Green– 2nd Cluster; Orange– 3rd Cluster; Red– 4th Cluster; Blue– 5th Cluster; Brown–None absent Cluster. (TIF) [file pone.0224920.s008.tif]

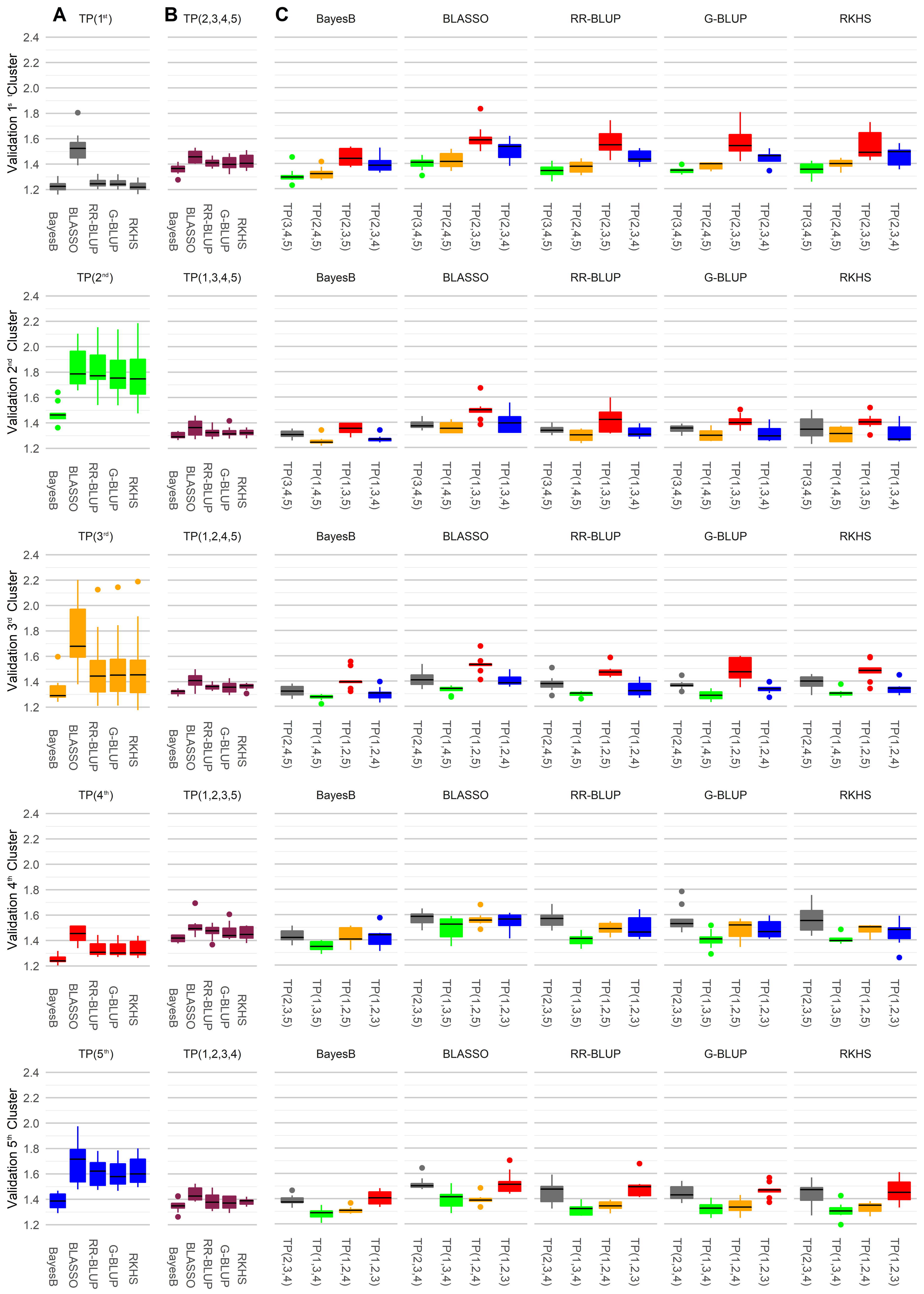

Supplement: S9 Fig — (A): Validation and training within clusters created by Discriminant Analysis of Principal Components (DAPC), informed in line. (B): Validation in DAPC line cluster and training population with all the remaining DAPC clusters. (C): Validation in DAPC line cluster and training with DAPC clusters column informed. Colors represent the absent cluster in training population. Black– 1st Cluster; Green– 2nd Cluster; Orange– 3rd Cluster; Red– 4th Cluster; Blue– 5th Cluster; Brown–None absent Cluster. (TIF) [file pone.0224920.s009.tif]
