# Supplementary material for: Cassava yield traits predicted by genomic selection methods
Source: PLoS One. 2019 Nov 14;14(11):e0224920. doi: 10.1371/journal.pone.0224920 (PMC6855463; doi:10.1371/journal.pone.0224920)
Supplement: S3 Table — (DOCX) [file pone.0224920.s012.docx]

**Table S3.** LRT analysis and Tukey’s pairwise test (p≤0.05) for prediction accuracies for the first cross validation strategy for fresh root yield, dry root yield and dry matter content.

| Deviance | DF | Fresh root yield | Dry root yield | Dry matter content |
| --- | --- | --- | --- | --- |
| Methods | 4 | 95.62* | 42.44* | 1,26 |
| Cross validation | 1 | 255.41* | 243.31* | 271.18* |
| Tukey multiple comparison test | | |  |  |
| BayesB |  | 0.9056 B (0.0922) | 0.9557 B (0.0866) | 0.9451 A (0.0826) |
| BLASSO |  | 0.9008 B (0.0945) | 0.9569 B (0.0859) | 0.9430 A (0.0813) |
| G-BLUP |  | 0.9054 B (0.0928) | 0.9461 C (0.0919) | 0.9449 A (0.0811) |
| RKHS |  | 0.9424 A (0.0895) | 0.9722 A (0.0896) | 0.9456 A (0.0829) |
| RR-BLUP |  | 0.9054 B (0.0929) | 0.9562 B (0.0870) | 0.9449 A (0.0811) |

*significance deviance for 5% probability in $\chi^{2}$ test. Major letters indicate significance differences between genomic prediction methods (p<0.05) by Tukey’s pairwise test. Standard error for the genomic prediction method is in parentheses.
