# Supplementary material for: Cassava yield traits predicted by genomic selection methods
Source: PLoS One. 2019 Nov 14;14(11):e0224920. doi: 10.1371/journal.pone.0224920 (PMC6855463; doi:10.1371/journal.pone.0224920)
Supplement: S4 Table — (DOCX) [file pone.0224920.s013.docx]

**Table S4.** LRT analysis and Tukey’s pairwise test (p≤0.05) of the prediction accuracies considering the 2^nd^, 3^rd^, and 4^th^ cross validation strategies to estimate the effect of population structure for fresh root yield, dry root yield and dry matter content.

| Deviance | DF | Fresh root yield | Dry root yield | Dry matter content |
| --- | --- | --- | --- | --- |
| Methods | 4 | 79.75* | 70.99* | 10.81* |
| Cross validation | 1 | 581.54* | 512.58* | 490.46* |
| Population structure | 1 | 1956.77* | 1716.17* | 1466.70* |
| Tukey multiple comparison test | | |  |  |
| BayesB |  | 0.1627 B (0.3507) | 0.2677 B (0.3105) | 0.4461 B (0.2468) |
| BLASSO |  | 0.1531 B (0.3443) | 0.2894 B (0.3237) | 0.4634 AB (0.2305) |
| G-BLUP |  | 0.1681 B (0.3480) | 0.2855 B (0.3122) | 0.4560 AB (0.2380) |
| RKHS |  | 0.2330 A (0.3329) | 0.3474 A (0.2938) | 0.4735 A (0.2278) |
| RR-BLUP |  | 0.1700 B (0.3439) | 0.2854 B (0.3192) | 0.4589 AB (0.2433) |
