# Supplementary material for: Cassava yield traits predicted by genomic selection methods
Source: PLoS One. 2019 Nov 14;14(11):e0224920. doi: 10.1371/journal.pone.0224920 (PMC6855463; doi:10.1371/journal.pone.0224920)
Supplement: S5 Table — (DOCX) [file pone.0224920.s014.docx]

**Table S5.** Different genomic selection Best linear unbiased prediction (BLUPs) scenarios due discriminant analysis of principal components (DAPC) cassava accessions clustering for fresh root yield, dry root yield and dry matter content.

| Cross-validation strategy | Populations – Clusters | | Population structure BLUPs | | | | | |
| --- | --- | --- | --- | --- | --- | --- | --- | --- |
|  |  |  | Fresh root yield | | Dry root yield | | Dry matter content | |
|  | Validation | Training | $r_{\hat{y}y}$ | *b* | $r_{\hat{y}y}$ | *b* | $r_{\hat{y}y}$ | *b* |
| 2^nd^ | 1 | 1* | 0.28 | 0.04 | 0.29 | 0.02 | 0.32 | -0.12 |
| 3^rd^ | 1 | All | -0.03 | 0.03 | -0.02 | 0.00 | -0.01 | -0.01 |
| 4^th^ | 1 | 3, 4 and 5 | 0.07 | 0.03 | 0.08 | -0.01 | -0.08 | -0.07 |
| 4^th^ | 1 | 2, 4 and 5 | -0.07 | -0.01 | -0.05 | -0.03 | 0.03 | -0.04 |
| 4^th^ | 1 | 2, 3 and 5 | -0.15 | 0.14 | -0.20 | 0.15 | 0.02 | 0.13 |
| 4^th^ | 1 | 2, 3 and 4 | 0.09 | -0.04 | 0.09 | -0.07 | -0.14 | 0.03 |
| 2^nd^ | 2 | 2* | 0.21 | 0.05 | 0.18 | 0.22 | 0.09 | 0.30 |
| 4^th^ | 2 | 3, 4 and 5 | -0.04 | 0.00 | -0.04 | -0.01 | -0.04 | -0.07 |
| 3^rd^ | 2 | All | -0.07 | 0.00 | -0.07 | -0.03 | -0.04 | -0.09 |
| 4^th^ | 2 | 1, 4 and 5 | -0.06 | 0.00 | -0.04 | -0.02 | -0.06 | -0.11 |
| 4^th^ | 2 | 1, 3 and 5 | -0.10 | 0.02 | -0.10 | 0.01 | 0.01 | 0.00 |
| 4^th^ | 2 | 1, 3 and 4 | -0.15 | -0.04 | -0.17 | -0.04 | -0.17 | -0.09 |
| 2^nd^ | 3 | 3 * | 0.36 | -0.04 | 0.32 | -0.04 | 0.18 | 0.12 |
| 4^th^ | 3 | 2, 4 and 5 | -0.14 | -0.01 | -0.06 | -0.02 | -0.04 | -0.04 |
| 4^th^ | 3 | 1, 4 and 5 | -0.01 | 0.01 | 0.06 | -0.04 | -0.06 | -0.12 |
| 3^rd^ | 3 | All | -0.13 | -0.05 | -0.05 | -0.06 | -0.02 | -0.05 |
| 4^th^ | 3 | 1, 2 and 5 | -0.14 | -0.03 | -0.12 | 0.00 | -0.07 | 0.06 |
| 4^th^ | 3 | 1, 2 and 4 | -0.21 | -0.12 | -0.14 | -0.13 | -0.05 | -0.07 |
| 2^nd^ | 4 | 4* | 0.37 | -0.06 | 0.27 | 0.01 | 0.34 | -0.08 |
| 4^th^ | 4 | 2, 3 and 5 | -0.20 | 0.14 | -0.18 | 0.15 | 0.07 | 0.12 |
| 4^th^ | 4 | 1, 3 and 5 | -0.08 | 0.01 | -0.11 | 0.01 | 0.00 | -0.01 |
| 4^th^ | 4 | 1, 2 and 5 | -0.11 | -0.02 | -0.12 | 0.01 | -0.14 | 0.07 |
| 3^rd^ | 4 | All | -0.14 | 0.02 | -0.12 | 0.02 | 0.06 | 0.05 |
| 4^th^ | 4 | 1, 2 and 3 | -0.15 | -0.04 | -0.14 | -0.05 | 0.07 | 0.06 |
| 2^nd^ | 5 | 5* | 0.19 | 0.24 | 0.15 | 0.32 | 0.19 | 0.16 |
| 4^th^ | 5 | 2, 3 and 4 | 0.07 | -0.05 | 0.07 | -0.08 | -0.04 | 0.03 |
| 4^th^ | 5 | 1, 3 and 4 | 0.06 | -0.03 | 0.03 | -0.05 | -0.23 | -0.09 |
| 4^th^ | 5 | 1, 2 and 4 | 0.02 | -0.11 | -0.01 | -0.12 | -0.04 | -0.07 |
| 4^th^ | 5 | 1, 2 and 3 | 0.13 | -0.03 | 0.10 | -0.04 | -0.07 | 0.05 |
| 3^rd^ | 5 | All | 0.11 | -0.06 | 0.09 | -0.08 | -0.06 | -0.03 |

$r_{\hat{y}y}$: predictive ability; *b*: bias; *Within DAPC cluster prediction.
